# Efficacy and safety study of targeted small-molecule drugs in the treatment of systemic lupus erythematosus

**DOI:** 10.1186/s13075-024-03331-8

**Published:** 2024-05-10

**Authors:** Shiheng Wang, Wanling Ning, Hanqing Tang, Chaochao Mu, Xiaosong Huang

**Affiliations:** 1 China Institute for History of Medicine and Medical Literature, China Academy of Chinese Medical Sciences, Beijing, 100700 China; 2 Hunan University of Chinese Medicine, Changsha Hunan, 410005 China; 3 School of Basic Medicine, Youjiang Medical University for Nationalities, Youjiang Guangxi, Baise, 533000 China; 4 Traditional Chinese Medicine Department, Tianjin Nankai District Bainian Renyitang Traditional Chinese Medicine Clinic, Tianjin, 301700 China; 5 Hunan Provincial Brain Hospital, Changsha Hunan, 410021 China

**Keywords:** SLE, Targeted small-molecule drugs, Safety, System evaluation, Bayesian network meta-analysis

## Abstract

**Background:**

Targeted small-molecule drugs in the treatment of systemic lupus erythematosus (SLE) have attracted increasing attention from clinical investigators. However, there is still a lack of evidence on the difference in the efficacy and safety of different targeted small-molecule drugs. Therefore, this study was conducted to assess the efficacy and safety of different targeted small-molecule drugs for SLE.

**Methods:**

Randomized controlled trials (RCTs) on targeted small-molecule drugs in the treatment of SLE in PubMed, Web of Science, Embase, and Cochrane Library were systematically searched as of April 25, 2023. Risk of bias assessment was performed for included studies using the Cochrane’s tool for evaluating the risk of bias. The primary outcome indicators were SRI-4 response, BICLA response, and adverse reaction. Because different doses and courses of treatment were used in the included studies, Bayesian network meta-regression was used to investigate the effect of different doses and courses of treatment on efficacy and safety.

**Results:**

A total of 13 studies were included, involving 3,622 patients and 9 targeted small-molecule drugs. The results of network meta-analysis showed that, in terms of improving SRI-4, Deucravacitinib was significantly superior to that of Baricitinib (RR = 1.32, 95% CI (1.04, 1.68), *P* < 0.05). Deucravacitinib significantly outperformed the placebo in improving BICLA response (RR = 1.55, 95% CI (1.20, 2.02), *P* < 0.05). In terms of adverse reactions, targeted small-molecule drugs did not significantly increase the risk of adverse events as compared to placebo (*P* > 0.05).

**Conclusion:**

Based on the evidence obtained in this study, the differences in the efficacy of targeted small-molecule drugs were statistically significant as compared to placebo, but the difference in the safety was not statistically significant. The dose and the course of treatment had little impact on the effect of targeted small-molecule drugs. Deucravacitinib could significantly improve BICLA response and SRI-4 response without significantly increasing the risk of AEs. Therefore, Deucravacitinib is very likely to be the best intervention measure. Due to the small number of included studies, more high-quality clinical evidence is needed to further verify the efficacy and safety of targeted small-molecule drugs for SLE.

**Supplementary Information:**

The online version contains supplementary material available at 10.1186/s13075-024-03331-8.

## Background

Systemic lupus erythematosus (SLE) is a systemic autoimmune disease induced by immune system dysfunction, which may cause damage to multiple organs and systems [1]. The pathogenesis of SLE is very complicated, involving almost all parts of the immune system. Multiple genetic, epigenetic, environmental, and hormonal factors may lead to the loss of self-tolerance and disorders of adaptive and innate immune systems [2]. Therefore, the treatment of SLE is complex and challenging.

At present, the treatment of SLE still relies mainly on glucocorticoids and immunosuppressants. However, the treatment efficacy is not satisfactory. Some patients are poorly responsive to these targeted small-molecule drugs, with relevant adverse reactions [3]. In recent years, with the in-depth understanding of the pathogenesis of SLE, it has been found that disordered production and abnormal expression or level of cytokines in SLE may be the main pathogenic factors. Disorders of cytokine network are conducive to inhibiting cytokine activity and promoting cytokine survival and the production of autoantibodies [4], which provides novel insights into the research and development of relevant new drugs. For example, such targeted small-molecule drugs as Janus Kinase Inhibitors(JAKs), Bruton Tyrosine Kinase (BTK), Spleen Tyrosine Kinase(SYK), and Sphingosine 1-Phosphate Receptor(Sphingosine 1-Phosphate Receptor) are being developed for the treatment of malignant and autoimmune diseases, including SLE [5]. These drugs differ from biological disease-modifying antirheumatic drugs (DMARDs). Some DMARDs are large-molecule drugs to be administered by injection, while small-molecule drugs are orally administered and can directly enter the cytoplasm to take effect. Due to such advantages as convenient administration, low production cost, and no immunogenicity, targeted small-molecule drugs have broad clinical application prospects [6].

Targeted small-molecule drugs are gaining increasing attention from clinical investigators. Many clinical trials have been published to investigate the efficacy and safety of targeted small-molecule drugs. However, the difference in their efficacy and safety is still unclear. In the meantime, there are a number of ongoing Randomized controlled trials (RCTs) (such as NCT03920267, NCT05620407, NCT05617677, NCT05672576 and NCT05648500) about targeted small-molecule drugs in the treatment of SLE on the clinical trial registration platform (https://beta.clinicaltrials.gov), indicating that the efficacy and safety of targeted small-molecule drugs in the treatment of SLE remain a research hotspot. Hence, in this study, the efficacy and safety of different targeted small-molecule drugs were compared by using Bayesian network meta-analysis based on published RCTs to find out the best targeted small-molecule drugs as much as possible, so as to provide an evidence-based reference for clinical application and study.

## Methods

### Study registration

This study was conducted in accordance with The Preferred Reporting Items for Systematic Reviews and Meta-Analyses for Network Meta-Analyses (PRISMA-NMA) and was prospectively registered in PROSPERO (ID: CRD42023420169).

### Eligibility criteria

#### Inclusion criteria

Population: Patients were definitely diagnosed with SLE. No restrictions were imposed on race, nationality, sex, age, and course of disease.

Intervention: Targeted small-molecule drugs (including JAKs, BTK, SYK, Proteasomes, Cereblon, and S1PR1) were used.

Comparison: Placebo.

Outcome:

(1) Primary outcome indicators: (1) Systemic Lupus Erythematosus Responder Index (SRI-4) response rate: A 24-item weighted score of lupus activity ranged from 0 to 105, with a higher score indicating higher disease activity and a score reduction of at least 4 points indicating being effective [7]; (2) BILAG-based Composite Lupus Assessment response(BICLA). The BILAG is used to assess the disease activity. If there is no Grade A or the number of Grade B is ≤ 1, and there is no deterioration in the disease activity (increased by less than 0.3 from baseline) according to physician’s global assessment (PGA), the drug is considered effective [8]; (3) Adverse reactions.

(2) Secondary outcome indicators: (1) CLASI-50: Cutaneous Lupus Erythematosus Disease Area and Severity Index (CLASI) is an indicator to evaluate the severity of cutaneous lupus erythematosus, and the score ranges from 0 to 70, with a higher score indicating more severe condition and higher activity, and a 50% reduction in the CLASI-A score called CLASI-50 [9]; (2) Swollen and tender joint count (baseline - end of treatment): It reflects the degree of joint pain and swelling, and a higher score indicates a more severe condition.

 Study design: RCTs.

#### Exclusion criteria

P (Population): Other types of SLE, such as lupus nephritis, active central nervous system lupus and lupus vasculitis.

I (Intervention): Different administration methods of targeted small-molecule drugs at the same dosage were used.

C: None.

O: None.

S (Study design): Meeting abstract published without peer review. If several studies were published based on the same RCT, a study with the largest sample size, the most complete follow-up time, and the most outcome indicators was included.

### Data sources and search strategy

RCTs of targeted small-molecule drugs in the treatment of SLE in PubMed, Embase, Cochrane Library, and Web of Science were systematically searched as of April 25, 2023 using the combination of subject words and free words. Search words included Systemic Lupus Erythematosus, Lupus Erythematosus Disseminatus, Libman-Sacks Disease, JAKs, Baricitinib, Agammaglobulinaemia Tyrosine Kinase, Fostamatinib. The search strategies are presented in Appendix 1.

### Study selection

We imported the retrieved literature into EndNoteX9 software and removed duplicate publications that were marked automatically and manually. As for the remaining literature, unqualified literature is deleted by reading the title and abstract; as for potentially qualified literature, the full text is downloaded and read to further screen the literature, so as to identify the original studies that are eligible for this systematic review. Literature screening was carried out independently by two investigators (Wang SH and Ning WL), and cross-check was conducted after completing the screening. Disagreements, if any, were discussed jointly with a third investigator (Tang HQ).

### Data extraction

A data extraction table was designed by two investigators (Wang SH and Zhang FX) according to the information required in the study, and data were extracted independently by them. The contents included ① Basic information: Title, author, year, study design, diagnosis criteria, intervention measures, course of treatment, and outcome indicators; ② Demographic characteristics: Sample size, age, and gender; ③ Methodological information: randomization method, allocation concealment protocol, blinding method, data integrity, selective reporting od results, other bias. If the information extracted by them was inconsistent, they would discuss the problem with each other to reach a consensus.

### Risk of bias in studies

Two investigators (Wang SH and Ning WL) used Cochrane’s tool for evaluating the risk of bias in RCTs [10] to assess the risk of bias in the included studies. This assessment tool included the following 7 items: generation of random sequences, allocation concealment, blinding of subjects and intervention providers, blinding of result evaluators, incomplete result data, selective reporting of results, and other sources of bias. Each item was graded as low-bias, high-bias, or unclear. The assessment results of the risk of bias were directly displayed using Revman5.4.

### Synthesis methods

A Bayesian random-effects model was used to compare the efficacy of various intervention measures. The Markov Chain Monte Carlo (MCMC) method was used for modeling, and 4 Markov chains were run at the same time. The number of annealing was set to 20,000, and the modeling was completed after 50,000 simulative iterations. Deviance information criterion (DIC) was used to compare model fitting and global consistency, and we would adopt the node-splitting method to analyze local consistency if closed loops existed. In addition, these intervention measures were ranked based on surface under the cumulative ranking curve (SUCRA), and the league table was generated to compare the difference in the effects among intervention measures.

Since the included studies were multi-arm studies on targeted small-molecule drugs with different doses and courses of treatment, Bayesian network meta-regression was adopted to analyze whether there were significant differences in efficacy and safety among targeted small-molecule drugs at different doses and courses of treatment in comparison with the placebo. When the number of included studies in the meta-analysis of an outcome indicator was ≥ 10, a funnel plot would be used to intuitively reflect the publication bias. The analysis was completed using Stata 15.0 (Stata Corporation, College Station, TX) and R4.2.0 (R development Core Team, Vienna, http://www.R-project.org). *P* < 0.05 indicates that the difference is statistically significant.

## Results

### Study selection

**Fig. 1:**
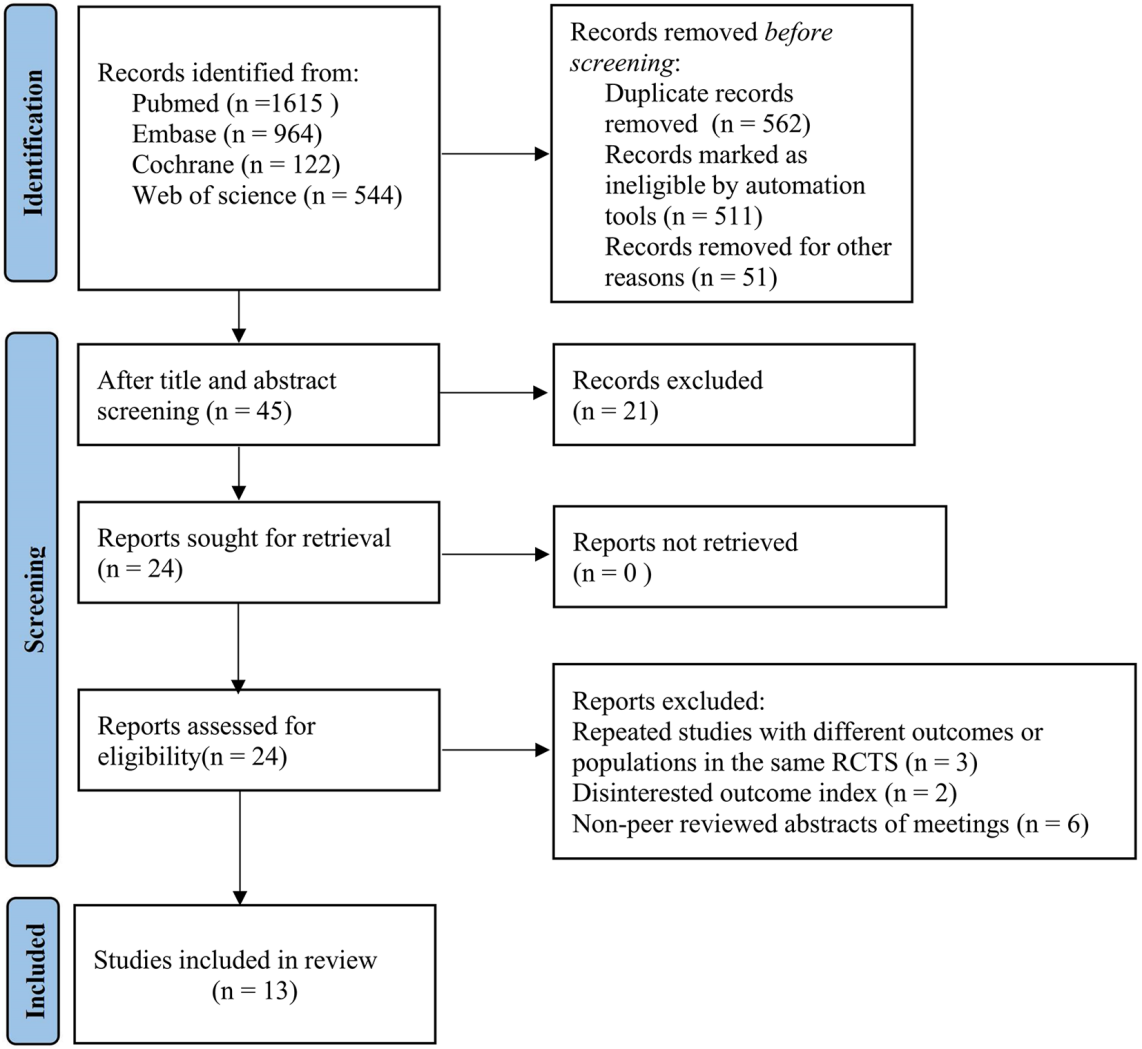
Literature screening process

Through a preliminary search, 3,245 relevant articles were obtained, and 562 duplicate articles were excluded. After reading the title and abstract, 24 articles were included, and 11 articles were included through full-text screening (3 studies of the same RCT repeatedly published with different outcomes or populations, 2 studies with no interest outcome indicators, and 6 conference abstracts without peer review). Finally, 13 studies were included [11–23]. The literature screening process is illustrated in Fig. 1.

### Study characteristics

Thirteen studies were included in this analysis, involving a total of 3,622 patients, and the authors of the included studies were from the United States, the United Kingdom, Germany, Australia, and Switzerland. The year of publication was between 2016 and 2023, and most of the studies were published in recent three years. The diagnosis criteria for SLE were mainly the American College of Rheumatology classification criteria [24, 25]. Eleven studies [11–19, 21–23] were multi-arm studies on a targeted small-molecule drug at different doses. Targeted small-molecule drugs used in the intervention group included JAKs (Baricitinib Filgotinib, Deucravacitinib, GSK2586184), BTK (Fenebrutinib, Evobrutinib), Cereblon (Iberdomide), SYK (Lanraplenib), and S1PR1 (Cenerimod). The basic characteristics of included studies are provided in Table 1.

**Table 1:** Basic characteristics of the original studies included in this systematic review

**No.**	**First author**	**Author’s country**	**Publication year**	**NCT No.**	**Diagnostic criteria for systemic lupus erythematosus (SLE)**		**Mean SLEDAI-2 K score(baseline)**
1	Thomas Dörner	Germany	2022	NCT02708095	American College of Rheumatology classification criteria OR the 2012 Systemic Lupus Erythematosus International Collaborating Clinics (SLICC) criteria		≥ 4
2	Eric Morand	Australia	2023	NCT03252587	American College of Rheumatology classification criteria		≥ 6,4
3	L Kahl	UK	2016	NCT01777256	American College of Rheumatology classification criteria		/
4	Michelle Petri	USA	2023	NCT03616964	American College of Rheumatology classification criteria		≥ 6, clinical ≥ 4
5	Peter E Lipsky	USA	2022	NCT03161483	American College of Rheumatology classification criteria		≥ 6
6	Joan T. Merrill	USA	2022	NCT03161483	American College of Rheumatology classification criteria		≥ 4
7	Eric Morand	Australia	2023	NCT03616912	American College of Rheumatology classification criteria		≥ 6
8	Daniel J. Wallace	USA	2023	NCT02975336	American College of Rheumatology classification criteria		≥ 6
9	Daniel J Wallace	USA	2018	/	American College of Rheumatology classification criteria		≥ 4
10	Victoria P. Werth	USA	2022	NCT03134222	CLASI		/
11	Richard A Furie	USA	2022	NCT02185040	American College of Rheumatology classification criteria		≥ 4
12	Viktoria Hermann	Switzerland	2019	NCT02472795	American College of Rheumatology classification criteria		≥ 2
13	David Isenberg	UK	2021	NCT02908100	American College of Rheumatology classification criteria		≥ 8
**No.**	**First author**	**Intervention methods (including dose)**	**Types of intervention drugs**	**Sample size**	**Gender (female)**	**Age(Mean(SD))**	**Course of treatment**
1	Thomas Dörner [11]	Baricitinib 2 mg Baricitinib 4 mg Placebo	JAK	105 104 105	96(91.4%) 99(95.2%) 99(94.3%)	43.2 (11.0) 45.0 (12.4) 44.9 (12.8)	24w
2	Eric Morand [12]	Deucravacitinib3 mg Deucravacitinib6 mg Deucravacitinib12 mg Placebo	TYK2	91 93 89 90	85(93.4%) 88(94.6%) 81(91.0%) 80(88.9%)	40.2 (11.9) 40.9 (12.5) 39.0 (10.6) 40.1 (13.1)	48w
3	L Kahl [13]	GSK2586184 50 mg GSK2586184 100 mg GSK2586184 200 mg GSK2586184 400 mg Placebo	JAK	9 10 10 10 11	9100%) 10(100%) 10(100%) 10(100%) 11(100%)	38.0 (12.55) 43.1 (11.23) 37.3 (7.15) 7.5 (10.99) 36.9 (10.14)	12w
4	Michelle Petri [14]	Baricitinib 2 mg Baricitinib 4 mg Placebo	JAK	261 258 256	246(94.3%) 245(94.9%) 241(94.1%)	42.8 (13.0) 42.2 (12.1) 43.5 (13.5)	52w
5	Peter E Lipsky [15]	Placebo Iberdomide 0.15 mg Iberdomide 0.3 mg/0.45 mg	Cereblon E3	83 42 82	/	/	24w
6	Joan T. Merrill [16]	Iberdomide 0.45 mg Iberdomide 0.30 mg Iberdomide 0.15 mg Placebo	Cereblon E3	81 82 42 83	79(97.5%) 77(93.9%) 41(97.6%) 81((97.6%)	46.4(11.2) 44.7(13.7) 43.8(13.0) 43.4(13.3)	24w
7	Eric Morand [17]	Placebo Baricitinib 2 mg Baricitinib 4 mg	JAK	253 255 252	237(93.6%) 238(93.3%) 237(94.0%)	42·0 (12.0) 42·9 (12.4) 41·5 (12.9)	52w
8	Daniel J. Wallace [18]	Placebo Evobrutinib 25 mg Evobrutinib 75 mg Evobrutinib 100 mg	BTK	117 118 117 117	110(94.0%) 112(94.9%) 111(94.9%) 112(95.7%)	40.2 (12.5) 38.8 (12.5) 41.5 (12.5) 42.2 (11.8)	52w
9	Daniel J Wallace [19]	Placebo Baricitinib 2 mg Baricitinib 4 mg	JAK	105 105 104	/	44·9 (12.8) 43·2 (11.0) 45·0 (12.4)	24w
10	Victoria P. Werth [20]	Placebo Lanraplenib 30 mg Filgotinib 200 mg	JAK	9 19 17	9(100%) 19(100%) 17(100%)	46 (7.3) 51 (9.0) 43 (11.5)	12w
11	Richard A Furie [21]	Placebo Iberdomide 0.3 mg Iberdomide 0.3 mg Iberdomide 0.6/0.3 mg Iberdomide 0.6 mg	Cereblon E3	8 8 8 9 9	7(87.5%) 8(100%) 7(87.5%) 8(88.9%) 9(100%)	44.8 (6.6) 46.0 (8.6) 48.0 (10.9) 49.8 (13.1) 47.2 (13.6)	12w
12	Viktoria Hermann [22]	Placebo Cenerimod 0.5 mg Cenerimod 1 mg Cenerimod 2 mg Cenerimod 4 mg	S1PR	17 12 12 13 13	16(94.1%) 11(91.7%) 12(100%) 12(92.3%) 10(76.9%)	41.0(9.5) 41.4(13.2) 37.0(6.4) 39.2(11.8) 41.7(8.1)	18w
13	David Isenberg [23]	Placebo fenebrutinib150 mg Fenebrutinib 400 mg	BTK	86 87 87	85(98.8%) 82(94.3%) 84(96.6%)	40(12.5) 44(13.5) 39(12.5)	48w

### Risk of bias in the included studies

**Fig. 2:**
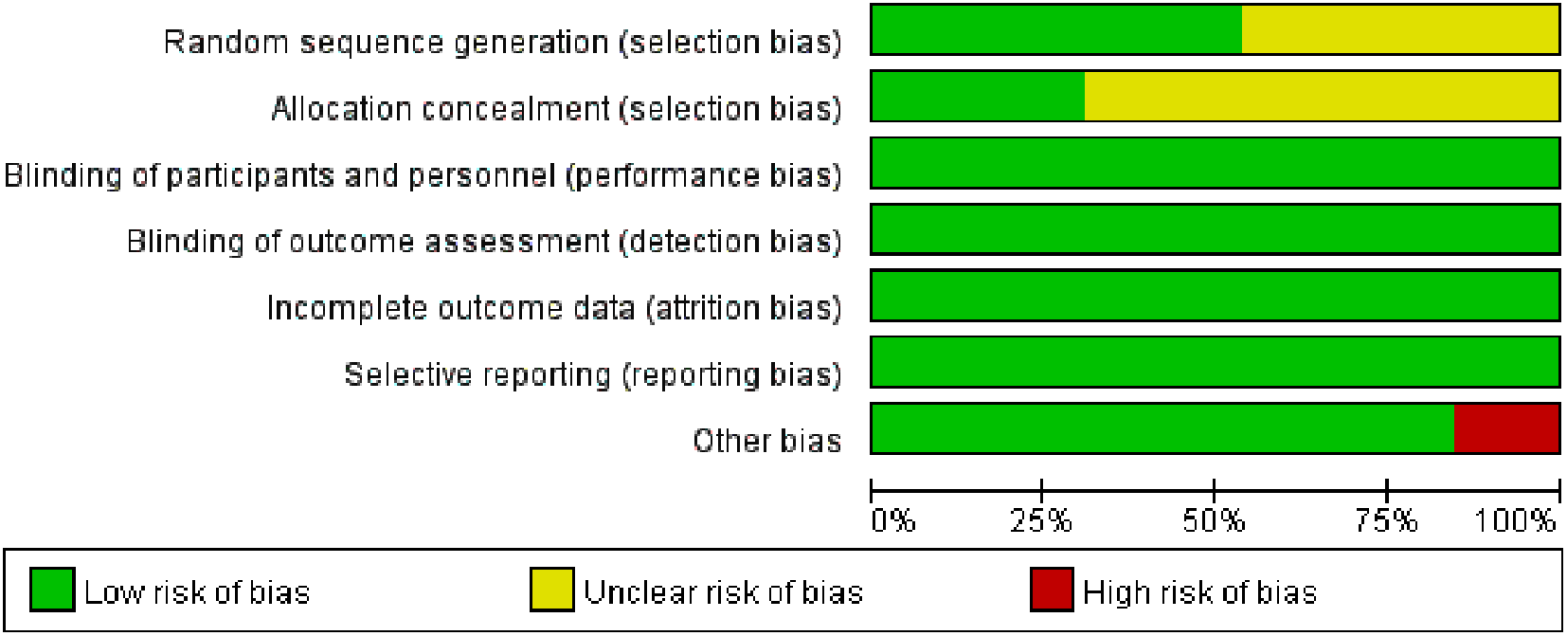
Risk of bias graph of all included studies

**Fig. 3:**
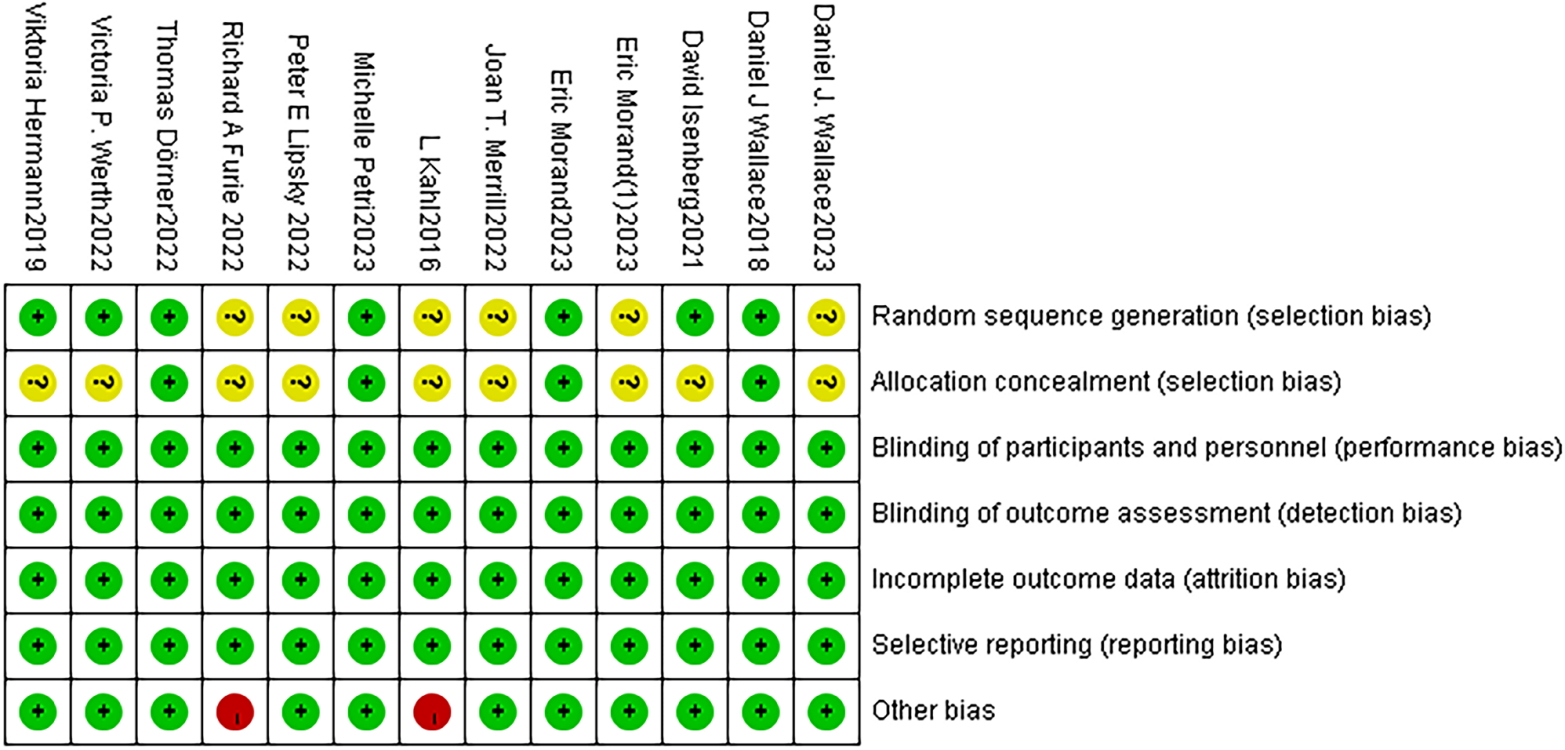
Risk of bias summary of all included studies

In terms of randomization methods, the computer-generated random sequence method was used in 4 studies [11, 14, 17, 18], and the inter-active web response system was used in 3 studies [19, 21, 22]. Allocation concealment was used in 4 studies [11, 14, 17, 18]. The blinding method was used in all studies. Data from all studies were complete, and no selective reporting was found. The sample size of 2 studies [13, 21] was small, which may cause publication bias. Figures 2 and 3 show the results of the risk of bias for each included study.

### Meta-analysis

#### SRI-4 response

##### The correlation among intervention measures

**Fig. 4:**
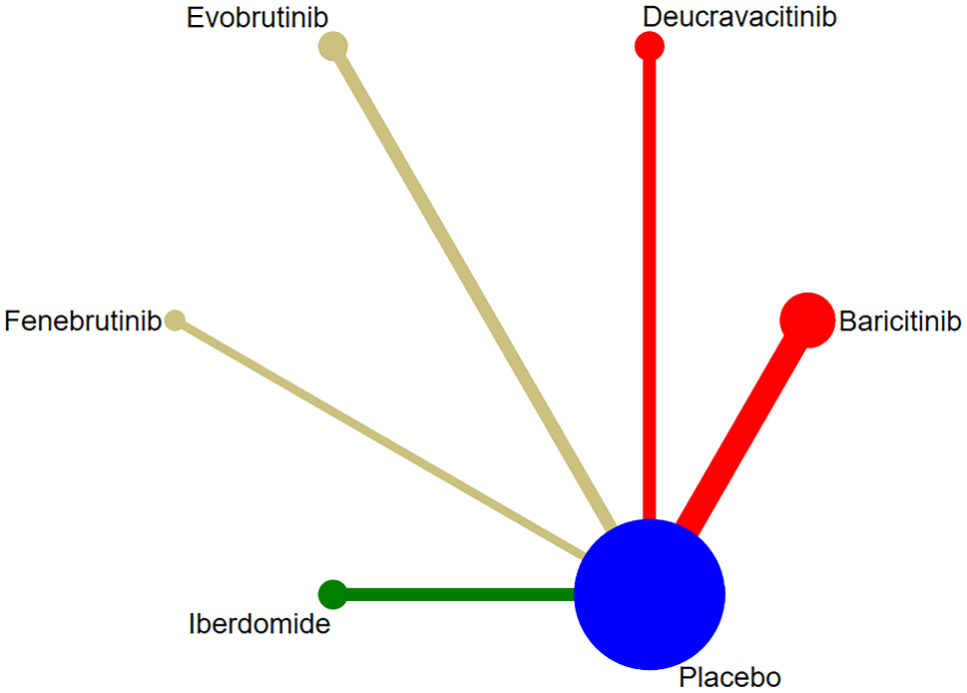
Network diagram of targeted drug therapy in the treatment of SLE using SRI-4 as the outcome indicator

SRI-4 response was reported in 7 studies [12, 14, 16–19, 23], involving 5 targeted small-molecule drugs from JAKs, BTK, and Cereblon. The comparison between targeted small-molecule drugs and the placebo was only reported in each study, and there was no pairwise comparison among targeted small-molecule drugs. The number of studies on direct comparison between Baricitinib and the placebo was the largest, and there was no closed loop, as shown in Fig. 4. (JAKs: red; BTK: khaki; Cereblon: green; SYK: yellow; S1PR1:purple; Placebo: blue).

##### Synthesized results

**Fig. 5:**
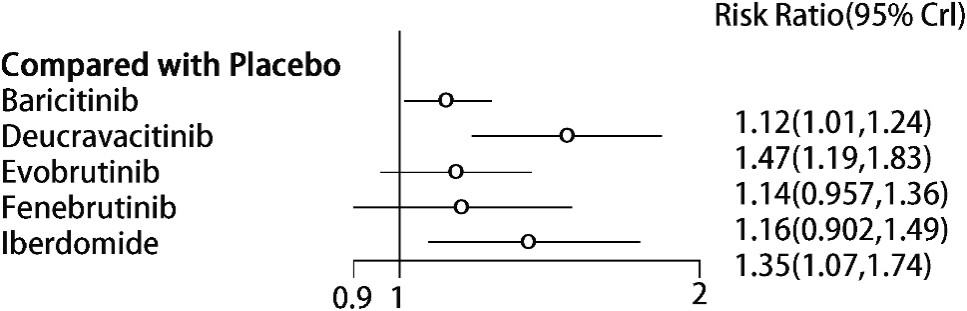
Forest plot of meta-analysis of SRI-4 response of targeted small-molecule drug therapy compared with placebo

Network meta-analysis results showed that 3 targeted small-molecule drugs (Baricitinib, Deucravacitinib, and Iberdomideb) were significantly superior to the placebo in improving SRI-4 response (*P* < 0.05) (Fig. 5). There were differences in efficacy among some targeted small-molecule drugs. The effect of Deucravacitinib was significantly superior to that of Baricitinib (RR = 1.32, 95% CI (1.04, 1.68), *P* < 0.05) (Table 2). The top three drugs in the SUCRA ranking were Deucravacitinib (0.91), Iberdomideb (0.79), and Fenebrutinib (0.45) (Table 3).

**Table 2:** League table of SRI-4 response of targeted small-molecule drug therapy

RR(95%CI)						
	Baricitinib	Deucravacitinib	Evobrutinib	Fenebrutinib	Iberdomide	Placebo
Baricitinib	0					
Deucravacitinib	**0.76 (0.59, 0.96)**	0				
Evobrutinib	0.98 (0.81, 1.20)	1.29 (0.98, 1.71)	0			
Fenebrutinib	0.97 (0.74, 1.26)	1.28 (0.91, 1.78)	0.989 (0.73, 1.33)	0		
Iberdomide	0.83 (0.63, 1.07)	1.09 (0.79, 1.51)	0.84(0.62, 1.12)	0.86 (0.59, 1.20)	0	
Placebo	**1.12 (1.01, 1.24)**	**1.47 (1.19, 1.83)**	1.14 (0.96, 1.36)	1.16 (0.90, 1.49)	**1.35 (1.07, 1.74)**	0

**Table 3:** Probability and ranking of Bayesian network meta-analysis of primary outcome indicators of each targeted small-molecule drug

Interventions	SRI-4 response		BICLA response		Adverse reactions	
	SUCRA	Rank	SUCRA	Rank	SUCRA	Rank
Baricitinib(JAKs)	0.38	6	0.40	3	0.61	4
Deucravacitinib(JAKs)	0.91	1	0.98	1	0.59	5
Evobrutinib(BTK)	0.43	5	/	/	0.44	6
Fenebrutinib(BTK)	0.45	3	0.56	2	0.30	8
Iberdomide(Cereblon)	0.79	2	/	/	0.13	10
Cenerimod(S1PR1)	/	/	/	/	0.93	1
Filgotinib(JAKs)	/	/	/	/	0.73	2
GSK2586184(JAKs)	/	/	/	/	0.29	9
Lanraplenib(SYK)	/	/	/	/	0.33	7
Placebo	0.43	4	0.07	4	0.66	3

##### Meta-regression

Because different doses of targeted small-molecule drugs and various courses of treatment were adopted in the included studies, meta-regression was performed on the dose and the course of treatment to discuss the effect of the dose and the course of treatment on SRI-4 response. The results showed that the efficacy and safety of targeted small-molecule drugs were not significantly correlated with the dose and the course of treatment as compared to the placebo, and the results were not statistically significant (Table 4).

**Table 4:** Meta-regression results of primary outcome indicators of each targeted small-molecule drug

Outcome indicators	Intervention measures	Dose (MD/RR(95%CI))	Course (MD/RR(95%CI))
SRI-4 response	Baricitinib	0.21 (-7.87, 5.70)	-0.21 (-0.61, 0.21)
	Deucravacitinib	-0.28 (-8.63, 3.61)	0.76 (-9.04, 21.90)
	Evobrutinib	-0.37 (-2.11, 1.12)	-2.63 (-28.21, 8.44)
	Fenebrutinib	0.02 (-0.65, 0.71)	-0.29 (-10.93, 6.41)
	Iberdomide	0.04 (-9.22, 17.68)	0.72 (-9.28, 10.93)
BICLA response	Baricitinib	0.27 (-5.25, 6.81)	-0.06 (-0.47, 0.35)
	Deucravacitinib	-0.51 (-6.04, 3.16)	0.41 (-7.12, 11.44)
	Fenebrutinib	-0.54 (-2.79, 1.16)	0.31 (-6.48, 9.64)
Adverse reactions	Baricitinib	0.11 (-7.71, 7.34)	-0.44 (-1.06, 0.18)
	Cenerimod	0.75 (-12.39, 14.43)	0.29 (-15.12, 12.64)
	Deucravacitinib	-2.24 (-15.03, 5.90)	-1.31 (-10.18, 5.44)
	Evobrutinib	-0.32 (-3.41, 2.63)	-0.64 (-8.74, 5.15)
	Fenebrutinib	-1.57 (-5.47, 1.63)	-0.46 (-8.92, 6.75)
	Filgotinib	0.57 (-6.22, 9.51)	-2.25 (-38.65, 12.87)
	GSK2586184	-0.52 (-1.81, 0.69)	0.76 (-6.33, 13.21)
	Iberdomide	0.97 (-4.88, 10.19)	-2.29 (-6.35, 0.90)
	Lanraplenib	0.31 (-17.59, 21.22)	0.43 (-13.30, 12.55)

#### BICLA response

##### The correlation among each intervention measure

**Fig. 6:**
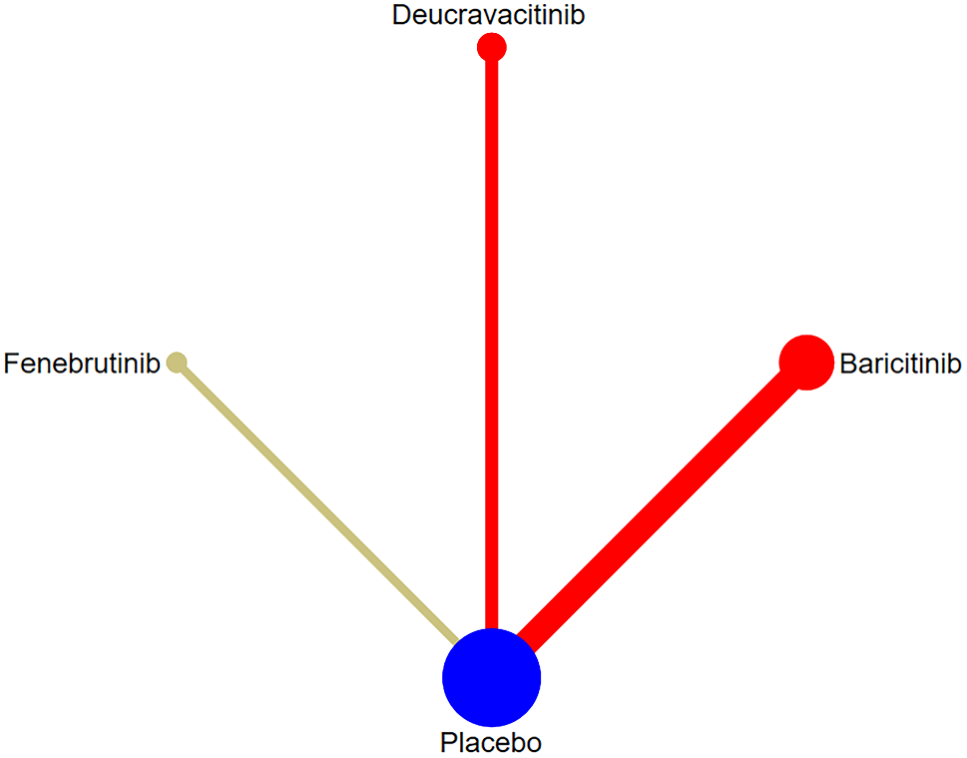
Network diagram of targeted drug therapy in the treatment of SLE using BICLA response as the outcome indicator

BICLA response was reported in 5 studies [12, 14, 17, 19, 23], involving 3 targeted small-molecule drugs from JAKs and BTK. The comparison between targeted small-molecule drugs and the placebo was only reported in each study, and there was no pairwise comparison among targeted small-molecule drugs. The number of studies directly comparing Baricitinib and the placebo was the greatest, and there was no closed loop, as shown in Fig. 6.

##### Synthesized results

**Fig. 7:**
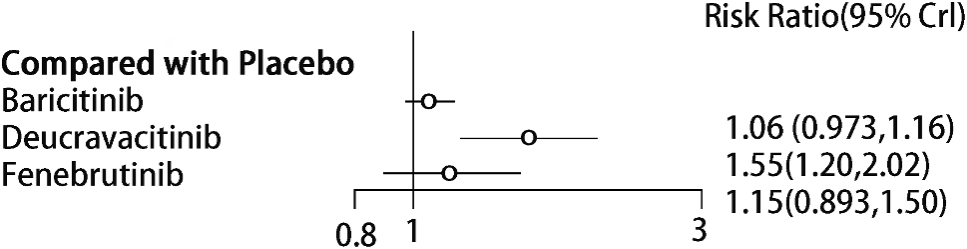
Forest plot of meta-analysis of BICLA response of targeted small-molecule drug therapy compared with placebo

Deucravacitinib significantly outperformed the placebo in improving BICLA response (RR = 1.55, 95% CI (1.20, 2.02), *P* < 0.05) (Fig. 7). There were differences in the efficacy among some targeted small-molecule drugs. Deucravacitinib was more effective than Baricitinib (RR = 1.47, 95% CI (1.11, 1.94), *P* < 0.05) (see Table 5). The top three drugs in the SUCRA ranking were Deucravacitinib (0.98), Fenebrutinib (0.56), and Baricitinib, in sequence (0.40) (Table 3).

**Table 5:** League table of BICLA stable response of targeted small-molecule drug therapy

RR(95%CI)				
	Baricitinib	Deucravacitinib	Fenebrutinib	Placebo
Baricitinib	0			
Deucravacitinib	**0.68 (0.52, 0.91)**	0		
Fenebrutinib	0.92 (0.69, 1.21)	1.35 (0.92, 1.95)	0	
Placebo	1.06 (0.97, 1.16)	**1.56 (1.19, 2.04)**	1.16 (0.89, 1.52)	0

##### Meta-regression

Due to differences in the dose of targeted small-molecule drugs and the course of treatment in the included studies, meta-regression was performed on the dose and the course of treatment to explore the effect of the dose and the course of treatment on BICLA response. The results showed that the efficacy and safety of targeted small-molecule drugs were not significantly correlated with the dose and the course of treatment as compared to the placebo, and the results were not statistically significant (Table 4).

#### Adverse reactions

##### The correlation among each intervention measure

**Fig. 8:**
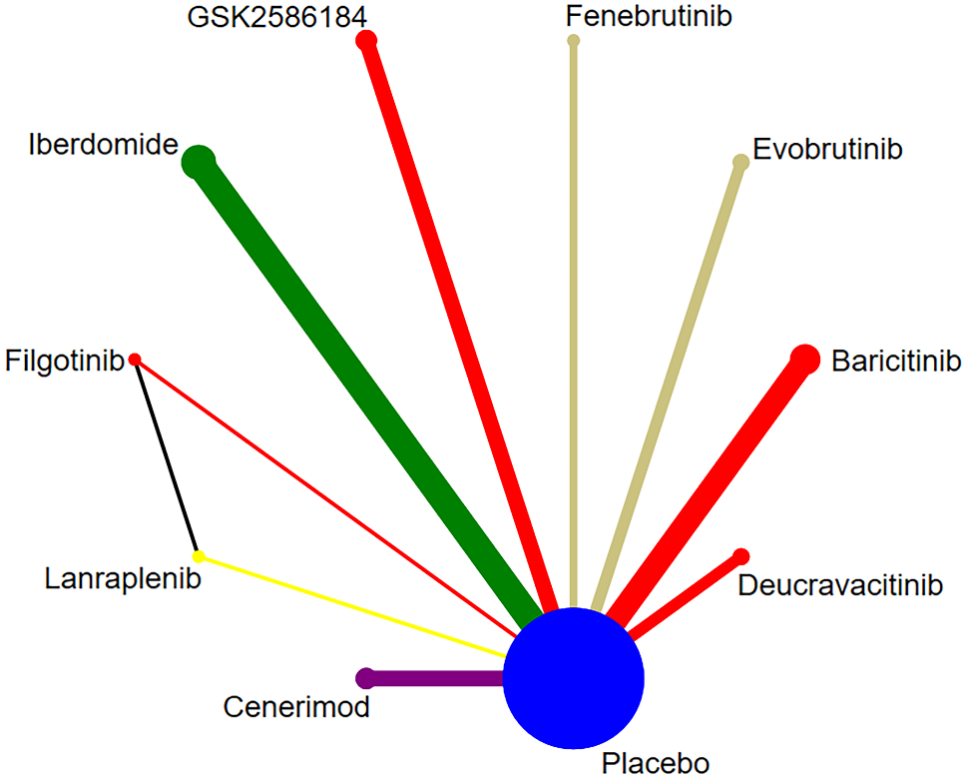
Network diagram of targeted drug therapy in the treatment of SLE using adverse reaction as the outcome indicator

Adverse reactions were reported in 11 studies [12–14, 16–23], involving 9 targeted small-molecule drugs from JAKs, BTK, SYK, Cereblon, and S1PR1. There was one closed loop (Filgotinib-Lanraplenib-Placebo). The studies directly comparing Iberdomide and the placebo were the largest in number, as shown in Fig. 8.

##### Synthesized results

**Fig. 9:**
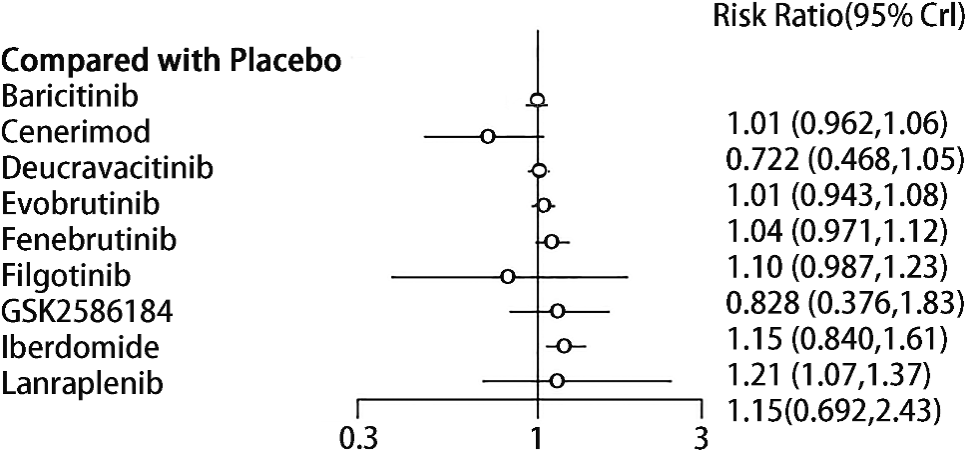
Forest plot of meta-analysis of adverse reaction of targeted small-molecule drug therapy compared with placebo

There were no statistically significant differences in the adverse reactions between targeted small-molecule drugs and the placebo. The safety profile of Iberdomide was significantly lower than that of placebo (RR = 1.21, 95% CI (1.07, 1.37), *P* < 0.05)(Fig. 9). There were differences in the adverse reactions among some targeted small-molecule drugs. Baricitinib (RR = 0.84, 95% CI (0.73, 0.95), *P* < 0.05), Cenerimod (RR = 0.60, 95% CI (0.39, 0.89), *P* < 0.05), Deucravacitinib (RR = 0.84, 95% CI (0.73, 0.96), *P* < 0.05), and Evobrutinib (RR = 0.87, 95% CI (0.75, 0.99), *P* < 0.05) had fewer adverse reactions in comparison with Iberdomide (Table 6). The top three drugs in the SUCRA ranking were Cenerimod (0.93), Filgotinib (0.73), and Placebo (0.66), in order (Table 3).

**Table 6:** League table of adverse reaction of targeted small-molecule drug therapy

RR(95%CI)										
	Baricitinib	Cenerimod	Deucravacitinib	Evobrutinib	Fenebrutinib	Filgotinib	GSK2586184	Iberdomide	Lanraplenib	Placebo
Baricitinib	0									
Cenerimod	1.39 (0.96, 2.16)	0								
Deucravacitinib	0.99 (0.92, 1.09)	0.72 (0.47, 1.04)	0							
Evobrutinib	0.96 (0.89, 1.05)	0.69 (0.45, 1.01)	0.97 (0.88, 1.07)	0						
Fenebrutinib	0.92 (0.81, 1.03)	**0.66 (0.42, 0.96)**	0.92 (0.80, 1.04)	0.95 (0.83, 1.08)	0					
Filgotinib	1.22 (0.55, 2.69)	0.86 (0.37, 2.01)	1.22 (0.55, 2.67)	1.26 (0.57, 2.74)	1.33 (0.59, 2.89)	0				
GSK2586184	0.87 (0.66, 1.21)	0.63 (0.38, 1.03)	0.88 (0.63, 1.20)	0.91 (0.64, 1.25)	0.95 (0.67, 1.32)	0.71 (0.30, 1.71)	0			
Iberdomide	**0.84(0.73, 0.95)**	**0.60 (0.39, 0.89)**	**0.84 (0.73, 0.96)**	**0.87 (0.75, 0.99)**	0.91 (0.76, 1.07)	0.68 (0.30, 1.52)	0.95、(0.69, 1.34)	0		
Lanraplenib	0.875 (0.414, 1.462)	0.612 (0.272, 1.205)	0.877 (0.416, 1.467)	0.91 (0.42, 1.52)	0.95 (0.45, 1.59)	0.70 (0.35, 1.19)	0.99 (0.43, 1.88)	1.04 (0.48, 1.75)	0	
Placebo	1.007 (0.962, 1.058)	0.722 (0.468, 1.046)	1.01 (0.943, 1.08)	1.04 (0.97, 1.12)	1.09 (0.98, 1.23)	0.82 (0.37, 1.82)	1.15 (0.84, 1.61)	**1.20 (1.07, 1.36)**	1.15 (0.69, 2.43)	0

##### Meta-regression

Owing to the differences in the dose of targeted small-molecule drugs and the course of treatment, meta-regression was performed on the dose and the course of treatment to investigate the effect of the dose and the course of treatment on adverse reactions. The results showed that the safety of targeted small-molecule drugs was not significantly correlated with the dose and the course of treatment as compared to the placebo, and the results were not statistically significant (Table 4).

### Secondary outcome indicators

Our study showed that in terms of CLASI-50, Deucravacitinib was significantly superior to the placebo (*P* < 0.05). Deucravacitinib was more significantly effective than Fenebrutinib. Deucravacitinib was more significantly effective than Iberdomide. In terms of tender joint count, Baricitinib and Deucravacitinib were significantly superior to the placebo (*P* < 0.05), and there was no significant difference in the efficacy among all targeted small-molecule drugs. No significant differences were observed in swollen joint count between all targeted small-molecule drugs and the placebo, or among targeted small-molecule drugs. The detailed analysis results are shown in Appendix 2.

## Discussion

Thirteen studies were included in this analysis, including 9 targeted small-molecule drugs: Baricitinib, Cenerimod, Deucravacitinib, Evobrutinib, Fenebrutinib, Filgotinib, GSK2586184, Iberdomide, and Lanraplenib. A total of 6 outcome indicators were studied. In terms of efficacy indicators, Baricitinib, Deucravacitinib, and Iberdomide significantly outperformed the placebo. The safety profile of Iberdomide was significantly lower than that of placebo. There were differences in the therapeutic effect among some targeted small-molecule drugs. The safety profile of Baricitinib, Cenerimod, Deucravacitinib, and Evobrutinib was higher than that of Iberdomide. The adverse reactions of targeted small-molecule drug therapy were mostly mild, with very few serious adverse reactions. Adverse reaction symptoms include nausea, vomiting, urinary tract infection, upper respiratory tract infection. It was reported that these symptoms can resolve spontaneously or be cured after treatment.

The meta-regression results of this study showed that doses and courses of treatment had no significant impact on the results. In terms of specific outcome indicators, there were differences in the efficacy among different targeted small-molecule drugs. Deucravacitinib could significantly improve BICLA response and SRI-4 response without significantly increasing the risk of AEs. Therefore, Deucravacitinib is very likely to be the best intervention measure. Although the meta-regression results showed that doses and courses of treatment had no significant effects on the results, this may differ from reality. Therefore, attention shall still be paid to the choice of doses and courses of treatment in clinical practice.

Deurefacitinib is a potent, highly selective, and allosteric small-molecule inhibitor of Tyrosine Kinase 2 (TYK2) that plays its role through a new mode of binding to the Janushomology2 (JH2) pseudokinase domain, so as to keep the kinase in an inactive state. It can block the downstream signaling of Interleukin-12(IL-12), Interleukin-23(IL-23), Interleukin-23(IL-10), and type I interferon (IFN). The selectivity of Deurefacitinib for the inhibition of TYK2 is 200 times greater than that of JAK1s/JAK3s inhibition, and even 3,000 times greater than JAK2s inhibition in the cell-based analysis [26, 27]. The trial by Catlett IM et al. [28] show that Deucravacitinib also strongly inhibits lymphopenia induced by Interferon α-2-a (IFN α-2-a). This finding is of great significance for the treatment of SLE and other autoimmune diseases. In the case of SLE, IFNα promotes the migration of lymphocytes into lymph nodes, thereby reducing lymphocyte counts in peripheral blood, and leading to lymphopenia [29, 30]. Furthermore, SLE is characterized by a highly elevated expression of interferon regulatory gene (IRG), which is considered to have important pathophysiological significance. Therefore, Deucravacitinib inhibits the development of two pathologic characteristics of SLE. Additionally, Deucravacitinib was reported to be generally well-tolerated at single and multiple administrations for up to 12 days. All AEs were mild to moderate, with no serious or severe AEs, which was consistent with the study results of Eric Morand [14]. This indicated that Deurefacitinib exhibits good efficacy and safety in the treatment of SLE and has great potential.

Previously published meta-analysis and network meta-analysis showed that the efficacy of Belimumab was superior to that of other targeted small-molecule drugs. Belimumab is a human immunoglobulin (Ig) G1k monoclonal antibody targeting BlyS [31]. For example, MengJun Tao et al. [32]. conducted a network meta-analysis to investigate the effects of 9 biological agents on SRI-4 response in patients with SLE. Their results showed that only Belimumab was more significantly effective than the placebo in the improvement of SRI-4 response (RR = 2.03, 95% CI (1.38, 3.00), *P* < 0.05), while other biological agents were not significantly superior to the placebo alone (*P* > 0.05). The study of Borba HH showed that [33] Belimumab was significantly superior to the placebo in the improvement of SRI-4 response (RR = 1.19, 95% CI (1.04, 1.37), *P* < 0.05). According to the results of our study, Deurefacitinib significantly outperformed the placebo in the improvement of SRI-4 response [RR = 1.117, 95% CI (1.01, 1.24)]. The improvement in SRI-4 response with Belimumab was more significant. In terms of safety and tolerability, the study of Borba HH showed that [33] there was no significant difference between Belimumab and the placebo (RR = 1.01, 95% CI (0.99, 1.04), *P* > 0.05), which was similar to the results of Singh JA [34] (RR = 0.87, 95% CI (0.68, 1.11), *P* > 0.05). However, due to the difference in the included articles, RCTs of direct comparison between Deurefacitinib and Belimumab shall be conducted to further verify their difference in efficacy.

Notably, the results based on four studies of Baricitinib in the treatment of SLE showed that Baricitinib had a better therapeutic effect than placebo, and there were no significant differences in the incidence of adverse events. However, one Phase III clinical study (NCT03616912) of Baricitinib in the treatment of SLE was terminated due to safety issues. This trial showed that acute myocardial infarction (AMI), pneumonia and other serious adverse events occurred in the Baricitinib group (58/507), which may warrant attention. It’s necessary to hold a cautious attitude towards the study results, and more trials are needed to further verify the safety of Baricitinib.

### Strengths and limitations

Our study has several strengths. At present, targeted small-molecule drugs in the treatment of SLE are a research hotspot, and there is still a lack of evidence on the difference in efficacy and safety among different targeted small-molecule drugs. This is the first network meta-analysis of targeted small-molecule drugs in the treatment of SLE to compare the difference in efficacy and safety among various targeted small-molecule drugs. Meanwhile, the effect of the dose and the course of treatment on efficacy and safety was also considered. In addition, strict inclusion/exclusion criteria were used to include only full-text RCTs. The included studies were published in high levels of journals, which contributes to generating high-quality evidence.

There are some limitations to our study. Due to the small number of included studies, the differences in efficacy evaluation criteria, patient characteristics, sample size, and selection of outcome indicators, as well as no description of the randomisation method and allocation concealment in some included studies, the level of evidence may be compromised. The number of original articles included for some outcome indicators was small, which may cause a bias in the results. Multiple Phase II clinical trials were included in this study. Due to their small sample size, the interpretation of results may be limited. Nevertheless, Phase II clinical trials have an important value in supplementing evidence-based evidence and shall not be excluded in systematic reviews due to its significance. There is a certain publication bias in the results, which may also affect the results. Therefore, more multi-center, high-quality RCTs with large sample sizes are needed to provide more evidence in the future.

## Conclusions

Based on the evidence from this study, Baricitinib, Deucravacitinib, and Iberdomide were significantly superior to the placebo. There were differences in the efficacy among different targeted small-molecule drugs. Deucravacitinib is likely to be the best intervention measure. No significant differences were observed in safety between targeted small-molecule drugs and the placebo. Meanwhile, the dose and the course of treatment had little effect on the efficacy and safety of targeted small-molecule drugs. Due to the small number of included studies, more high-quality RCTs are needed to further verify the efficacy and safety of targeted small-molecule drugs.

### Electronic supplementary material

Below is the link to the electronic supplementary material.

Supplementary Material 1

Supplementary Material 2

## Data Availability

Data sharing not applicable to this article as no datasets were generated or analysed during the current study.

## Abbreviations

SLE: Systemic Lupus Erythematosus
RCTs: Randomized controlled trials
SRI-4: Systemic Lupus Erythematosus Responder Index response rate
BICLA: BILAG-based Composite Lupus Assessment
CLASI: Cutaneous Lupus Erythematosus Disease Area and Severity Index
