# Supplementary material for: Efficacy and safety study of targeted small-molecule drugs in the treatment of systemic lupus erythematosus
Source: Arthritis Res Ther. 2024 May 10;26:98. doi: 10.1186/s13075-024-03331-8 (PMC11083747; doi:10.1186/s13075-024-03331-8)
Supplement: Supplementary file 1 — Supplementary Material 1 [file 13075_2024_3331_MOESM1_ESM.doc]

1. **Search strategy**

| **Search number** | **Query** | **number** |
| --- | --- | --- |
| 1 | Lupus Erythematosus, Systemic[MeSH Terms] | 66,625 |
| 2 | (((((((((((((Lupus Erythematosus, Systemic[Title/Abstract]) OR (Systemic Lupus Erythematosus[Title/Abstract])) OR (Lupus Erythematosus Disseminatus[Title/Abstract])) OR (Libman-Sacks Disease[Title/Abstract])) OR (Libman Sacks Disease[Title/Abstract])) OR (disseminated lupus[Title/Abstract])) OR (erythematodes visceralis[Title/Abstract])) OR (lupovisceritis[Title/Abstract])) OR (lupus erythematodes disseminates[Title/Abstract])) OR (lupus erythematosus disseminates[Title/Abstract])) OR (lupus erythematosus visceralis[Title/Abstract])) OR (osler libman sacks disease[Title/Abstract])) OR (systemic lupus erythematodes[Title/Abstract])) OR (dermatovisceritism, malignant[Title/Abstract]) | 495,162 |
| 3 | (Lupus Erythematosus, Systemic[MeSH Terms]) OR ((((((((((((((Lupus Erythematosus, Systemic[Title/Abstract]) OR (Systemic Lupus Erythematosus[Title/Abstract])) OR (Lupus Erythematosus Disseminatus[Title/Abstract])) OR (Libman-Sacks Disease[Title/Abstract])) OR (Libman Sacks Disease[Title/Abstract])) OR (disseminated lupus[Title/Abstract])) OR (erythematodes visceralis[Title/Abstract])) OR (lupovisceritis[Title/Abstract])) OR (lupus erythematodes disseminates[Title/Abstract])) OR (lupus erythematosus disseminates[Title/Abstract])) OR (lupus erythematosus visceralis[Title/Abstract])) OR (osler libman sacks disease[Title/Abstract])) OR (systemic lupus erythematodes[Title/Abstract])) OR (dermatovisceritism, malignant[Title/Abstract])) | 516,689 |
| 4 | Janus kinases[MeSH Terms] | 14,763 |
| 5 | ((((((((Janus kinases[Title/Abstract]) OR (JAK Kinases[Title/Abstract])) OR (Janus Kinase[Title/Abstract])) OR (Tofacitinib[Title/Abstract])) OR (Solcitinib[Title/Abstract])) OR (Filgotinib[Title/Abstract])) OR (Baricitinib[Title/Abstract])) OR (Deucravacitinib[Title/Abstract])) OR (Beprocitinib[Title/Abstract]) | 12,599 |
| 6 | (Janus kinases[MeSH Terms]) OR (((((((((Janus kinases[Title/Abstract]) OR (JAK Kinases[Title/Abstract])) OR (Janus Kinase[Title/Abstract])) OR (Tofacitinib[Title/Abstract])) OR (Solcitinib[Title/Abstract])) OR (Filgotinib[Title/Abstract])) OR (Baricitinib[Title/Abstract])) OR (Deucravacitinib[Title/Abstract])) OR (Beprocitinib[Title/Abstract])) | 22,521 |
| 7 | Agammaglobulinaemia Tyrosine Kinase[MeSH Terms] | 2,172 |
| 8 | ((((((((Agammaglobulinaemia Tyrosine Kinase[Title/Abstract]) OR (Bruton's Tyrosine Kinase[Title/Abstract])) OR (Bruton Tyrosine Kinase[Title/Abstract])) OR (Brutons Tyrosine Kinase[Title/Abstract])) OR (B Cell Progenitor Kinase[Title/Abstract])) OR (Ibrutinib[Title/Abstract])) OR (Fenebrutinib[Title/Abstract])) OR (Evobrutinib[Title/Abstract])) OR (Orelabrutinib[Title/Abstract]) | 5,150 |
| 9 | (Agammaglobulinaemia Tyrosine Kinase[MeSH Terms]) OR (((((((((Agammaglobulinaemia Tyrosine Kinase[Title/Abstract]) OR (Bruton's Tyrosine Kinase[Title/Abstract])) OR (Bruton Tyrosine Kinase[Title/Abstract])) OR (Brutons Tyrosine Kinase[Title/Abstract])) OR (B Cell Progenitor Kinase[Title/Abstract])) OR (Ibrutinib[Title/Abstract])) OR (Fenebrutinib[Title/Abstract])) OR (Evobrutinib[Title/Abstract])) OR (Orelabrutinib[Title/Abstract])) | 5,683 |
| 10 | Syk Kinase[MeSH Terms] | 2,547 |
| 11 | ((((Syk Kinase[Title/Abstract]) OR (SYK Tyrosine Kinase[Title/Abstract])) OR (Spleen Tyrosine Kinase[Title/Abstract])) OR (Fostamatinib[Title/Abstract])) OR (Lanraplenib[Title/Abstract]) | 1,830 |
| 12 | (Syk Kinase[MeSH Terms]) OR (((((Syk Kinase[Title/Abstract]) OR (SYK Tyrosine Kinase[Title/Abstract])) OR (Spleen Tyrosine Kinase[Title/Abstract])) OR (Fostamatinib[Title/Abstract])) OR (Lanraplenib[Title/Abstract])) | 3,307 |
| 13 | (((((((((((Proteasomes[Title/Abstract]) OR (Carfilzomib[Title/Abstract])) OR (Ixazomib[Title/Abstract])) OR (Zetomipzomib[Title/Abstract])) OR (Cereblon[Title/Abstract])) OR (Lenalidomide[Title/Abstract])) OR (Iberdomide[Title/Abstract])) OR (Mezigdomide[Title/Abstract])) OR (S1PR1[Title/Abstract])) OR (Ozanimod[Title/Abstract])) OR (Amiselimod[Title/Abstract])) OR (Cenerimod[Title/Abstract]) | 11,447 |
| 14 | ((((Janus kinases[MeSH Terms]) OR (((((((((Janus kinases[Title/Abstract]) OR (JAK Kinases[Title/Abstract])) OR (Janus Kinase[Title/Abstract])) OR (Tofacitinib[Title/Abstract])) OR (Solcitinib[Title/Abstract])) OR (Filgotinib[Title/Abstract])) OR (Baricitinib[Title/Abstract])) OR (Deucravacitinib[Title/Abstract])) OR (Beprocitinib[Title/Abstract]))) OR ((Agammaglobulinaemia Tyrosine Kinase[MeSH Terms]) OR (((((((((Agammaglobulinaemia Tyrosine Kinase[Title/Abstract]) OR (Bruton's Tyrosine Kinase[Title/Abstract])) OR (Bruton Tyrosine Kinase[Title/Abstract])) OR (Brutons Tyrosine Kinase[Title/Abstract])) OR (B Cell Progenitor Kinase[Title/Abstract])) OR (Ibrutinib[Title/Abstract])) OR (Fenebrutinib[Title/Abstract])) OR (Evobrutinib[Title/Abstract])) OR (Orelabrutinib[Title/Abstract])))) OR ((Syk Kinase[MeSH Terms]) OR (((((Syk Kinase[Title/Abstract]) OR (SYK Tyrosine Kinase[Title/Abstract])) OR (Spleen Tyrosine Kinase[Title/Abstract])) OR (Fostamatinib[Title/Abstract])) OR (Lanraplenib[Title/Abstract])))) OR ((((((((((((Proteasomes[Title/Abstract]) OR (Carfilzomib[Title/Abstract])) OR (Ixazomib[Title/Abstract])) OR (Zetomipzomib[Title/Abstract])) OR (Cereblon[Title/Abstract])) OR (Lenalidomide[Title/Abstract])) OR (Iberdomide[Title/Abstract])) OR (Mezigdomide[Title/Abstract])) OR (S1PR1[Title/Abstract])) OR (Ozanimod[Title/Abstract])) OR (Amiselimod[Title/Abstract])) OR (Cenerimod[Title/Abstract])) | 42,230 |
| 15 | ((Lupus Erythematosus, Systemic[MeSH Terms]) OR ((((((((((((((Lupus Erythematosus, Systemic[Title/Abstract]) OR (Systemic Lupus Erythematosus[Title/Abstract])) OR (Lupus Erythematosus Disseminatus[Title/Abstract])) OR (Libman-Sacks Disease[Title/Abstract])) OR (Libman Sacks Disease[Title/Abstract])) OR (disseminated lupus[Title/Abstract])) OR (erythematodes visceralis[Title/Abstract])) OR (lupovisceritis[Title/Abstract])) OR (lupus erythematodes disseminates[Title/Abstract])) OR (lupus erythematosus disseminates[Title/Abstract])) OR (lupus erythematosus visceralis[Title/Abstract])) OR (osler libman sacks disease[Title/Abstract])) OR (systemic lupus erythematodes[Title/Abstract])) OR (dermatovisceritism, malignant[Title/Abstract]))) AND (((((Janus kinases[MeSH Terms]) OR (((((((((Janus kinases[Title/Abstract]) OR (JAK Kinases[Title/Abstract])) OR (Janus Kinase[Title/Abstract])) OR (Tofacitinib[Title/Abstract])) OR (Solcitinib[Title/Abstract])) OR (Filgotinib[Title/Abstract])) OR (Baricitinib[Title/Abstract])) OR (Deucravacitinib[Title/Abstract])) OR (Beprocitinib[Title/Abstract]))) OR ((Agammaglobulinaemia Tyrosine Kinase[MeSH Terms]) OR (((((((((Agammaglobulinaemia Tyrosine Kinase[Title/Abstract]) OR (Bruton's Tyrosine Kinase[Title/Abstract])) OR (Bruton Tyrosine Kinase[Title/Abstract])) OR (Brutons Tyrosine Kinase[Title/Abstract])) OR (B Cell Progenitor Kinase[Title/Abstract])) OR (Ibrutinib[Title/Abstract])) OR (Fenebrutinib[Title/Abstract])) OR (Evobrutinib[Title/Abstract])) OR (Orelabrutinib[Title/Abstract])))) OR ((Syk Kinase[MeSH Terms]) OR (((((Syk Kinase[Title/Abstract]) OR (SYK Tyrosine Kinase[Title/Abstract])) OR (Spleen Tyrosine Kinase[Title/Abstract])) OR (Fostamatinib[Title/Abstract])) OR (Lanraplenib[Title/Abstract])))) OR ((((((((((((Proteasomes[Title/Abstract]) OR (Carfilzomib[Title/Abstract])) OR (Ixazomib[Title/Abstract])) OR (Zetomipzomib[Title/Abstract])) OR (Cereblon[Title/Abstract])) OR (Lenalidomide[Title/Abstract])) OR (Iberdomide[Title/Abstract])) OR (Mezigdomide[Title/Abstract])) OR (S1PR1[Title/Abstract])) OR (Ozanimod[Title/Abstract])) OR (Amiselimod[Title/Abstract])) OR (Cenerimod[Title/Abstract]))) | 1,615 |

PubMed

| **Search number** | **Query** | **number** |
| --- | --- | --- |
| 1 | 'systemic lupus erythematosus'/exp | 114933 |
| 2 | 'protein kinase syk'/exp | 4703 |
| 3 | 'syk kinase'/exp OR 'syk kinase' OR (syk AND ('kinase'/exp OR kinase)) OR 'syk tyrosine kinase':ab,ti OR 'spleen tyrosine kinase':ab,ti OR 'protein kinase syk':ab,ti OR fostamatinib:ab,ti OR lanraplenib:ab,ti | 7394 |
| 4 | #10 OR #11 | 7394 |
| 5 | proteasomes OR carfilzomib:ab,ti OR ixazomib:ab,ti OR zetomipzomib:ab,ti OR cereblon:ab,ti OR lenalidomide:ab,ti OR iberdomide:ab,ti OR mezigdomide:ab,ti OR s1pr1:ab,ti OR ozanimod:ab,ti OR amiselimod:ab,ti OR cenerimod:ab,ti | 24681 |
| 6 | #6 OR #9 OR #12 OR #13 | 74903 |
| 7 | #3 AND #14 | 964 |

Embase

| **Search number** | **Query** | **number** |
| --- | --- | --- |
| 1 | ((((((((((((TS=(Systemic Lupus Erythematosus )) OR TS=(Lupus Erythematosus Disseminatus)) OR TS=(Libman-Sacks Disease)) OR TS=(Libman Sacks Disease)) OR TS=(disseminated lupus)) OR TS=(erythematodes visceralis)) OR TS=(lupovisceritis)) OR TS=(lupus erythematodes disseminates)) OR TS=(lupus erythematosus disseminates)) OR TS=(lupus erythematosus visceralis)) OR TS=(osler libman sacks disease)) OR TS=(systemic lupus erythematodes)) OR TS=(dermatovisceritism, malignant) | 63520 |
| 2 | (((((((((((((TS=(Janus kinases))) OR TS=(JAK Kinases)) OR TS=(Janus Kinase)) OR TS=(Tofacitinib)) OR TS=(Solcitinib)) OR TS=(Filgotinib)) OR TS=(Solcitinib)) OR TS=(Baricitinib)) OR TS=(Deucravacitinib)) OR TS=(Beprocitinib)) OR TS=(Agammaglobulinaemia Tyrosine Kinase)) OR TS=(Bruton's Tyrosine Kinase)) OR TS=(Bruton Tyrosine Kinase) OR TS=(Brutons Tyrosine Kinase) OR TS=(B Cell Progenitor Kinase) OR TS=(B-cell progenitor kinase) OR TS=(Ibrutinib) OR TS=(Fenebrutinib) OR TS=(Evobrutinib) OR TS=(Orelabrutinib) OR TS=(Syk Kinase) OR TS=(SYK Tyrosine Kinase) OR TS=(Spleen Tyrosine Kinase) OR TS=(protein kinase syk) OR TS=(Fostamatinib) OR TS=(Lanraplenib) OR TS=(Proteasomes) OR TS=(Carfilzomib) OR TS=(Ixazomib) OR TS=(Zetomipzomib) OR TS=(Cereblon) OR TS=(Lenalidomide) OR TS=(Iberdomide) OR TS=(Mezigdomide) OR TS=(S1PR1) OR TS=(Ozanimod) OR TS=(Amiselimod) OR TS=(Cenerimod) | 50243 |
| 3 | 1 AND 2 | 544 |

Web of science

| **ID** | **Search** | **Hits** |
| --- | --- | --- |
| #1 | MeSH descriptor: [Lupus Erythematosus, Systemic] explode all trees | 1420 |
| #2 | (Lupus Erythematosus, Systemic):ti,ab,kw OR (Systemic Lupus Erythematosus):ti,ab,kw OR (Lupus Erythematosus Disseminatus):ti,ab,kw OR (Libman-Sacks Disease):ti,ab,kw OR (Libman Sacks Disease):ti,ab,kw | 2836 |
| #3 | (disseminated lupus):ti,ab,kw OR (erythematodes visceralis):ti,ab,kw OR (lupovisceritis):ti,ab,kw OR (lupus erythematodes disseminates):ti,ab,kw OR (lupus erythematosus disseminates):ti,ab,kw | 16 |
| #4 | (lupus erythematosus visceralis):ti,ab,kw OR (osler libman sacks disease):ti,ab,kw OR (systemic lupus erythematodes):ti,ab,kw OR (dermatovisceritism, malignant):ti,ab,kw | 12 |
| #5 | #1 OR #2 OR #3 OR #4 | 3055 |
| #6 | MeSH descriptor: [Janus Kinases] explode all trees | 202 |
| #7 | (Janus kinases):ti,ab,kw OR (JAK Kinases):ti,ab,kw OR (Janus Kinase):ti,ab,kw OR (Tofacitinib):ti,ab,kw OR (Solcitinib):ti,ab,kw | 2341 |
| #8 | (Filgotinib):ti,ab,kw OR (Baricitinib):ti,ab,kw OR (Deucravacitinib):ti,ab,kw OR (Beprocitinib):ti,ab,kw | 1024 |
| #9 | #6 OR #7 OR #8 | 2918 |
| #10 | MeSH descriptor: [Agammaglobulinaemia Tyrosine Kinase] explode all trees | 35 |
| #11 | (Agammaglobulinaemia Tyrosine Kinase):ti,ab,kw OR (Bruton's Tyrosine Kinase):ti,ab,kw OR (Bruton Tyrosine Kinase):ti,ab,kw OR (Brutons Tyrosine Kinase):ti,ab,kw OR (B Cell Progenitor Kinase):ti,ab,kw | 520 |
| #12 | (B-cell progenitor kinase):ti,ab,kw OR (Ibrutinib):ti,ab,kw OR (Fenebrutinib):ti,ab,kw OR (Evobrutinib):ti,ab,kw OR (Orelabrutinib):ti,ab,kw | 856 |
| #13 | #10 OR #11 OR #12 | 1038 |
| #14 | MeSH descriptor: [Syk Kinase] explode all trees | 25 |
| #15 | (Syk Kinase):ti,ab,kw OR (SYK Tyrosine Kinase):ti,ab,kw OR (Spleen Tyrosine Kinase):ti,ab,kw OR (protein kinase syk):ti,ab,kw OR (Fostamatinib):ti,ab,kw | 202 |
| #16 | (Lanraplenib):ti,ab,kw | 6 |
| #17 | #14 OR #15 OR #16 | 205 |
| #18 | (Proteasomes):ti,ab,kw OR (Carfilzomib):ti,ab,kw OR (Ixazomib):ti,ab,kw OR (Zetomipzomib):ti,ab,kw OR (Cereblon):ti,ab,kw | 779 |
| #19 | (Lenalidomide):ti,ab,kw OR (Iberdomide):ti,ab,kw OR (Mezigdomide):ti,ab,kw OR (S1PR1):ti,ab,kw OR (Ozanimod):ti,ab,kw | 2742 |
| #20 | (Amiselimod):ti,ab,kw OR (Cenerimod):ti,ab,kw | 41 |
| #21 | #18 OR #19 OR #20 | 3102 |
| #22 | #9 OR #13 OR #17 OR #21 | 7150 |
| #23 | #5 AND #22 | 122 |

Cochrane library

1. **SUCRASorting diagram**

**
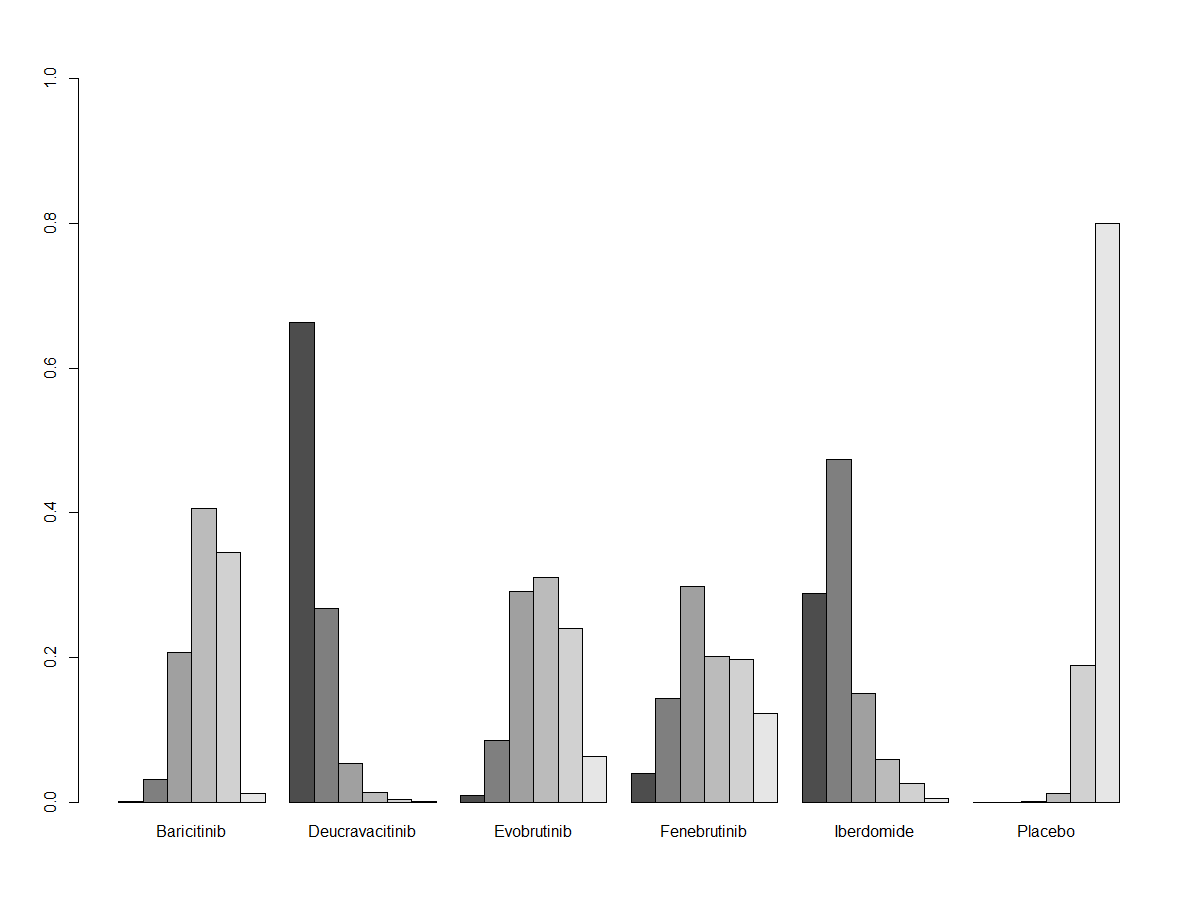
**

SRI-4 response

**
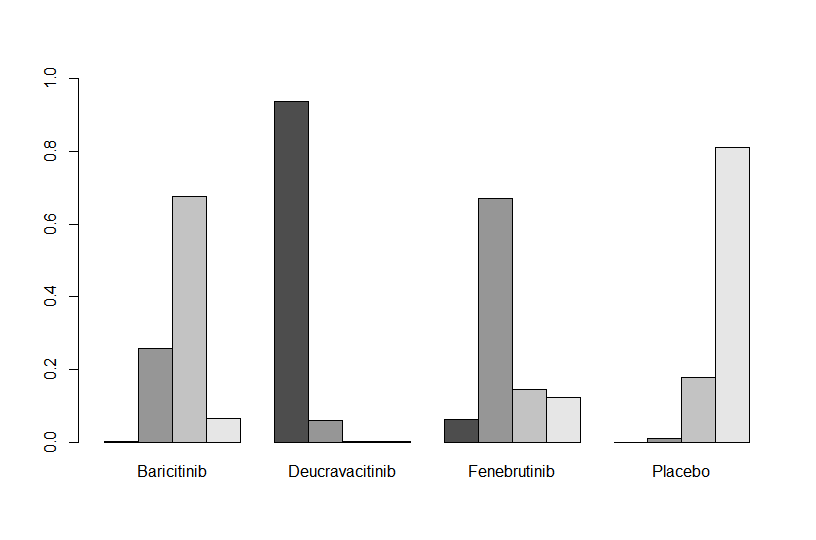
**

BICLA reponse

**
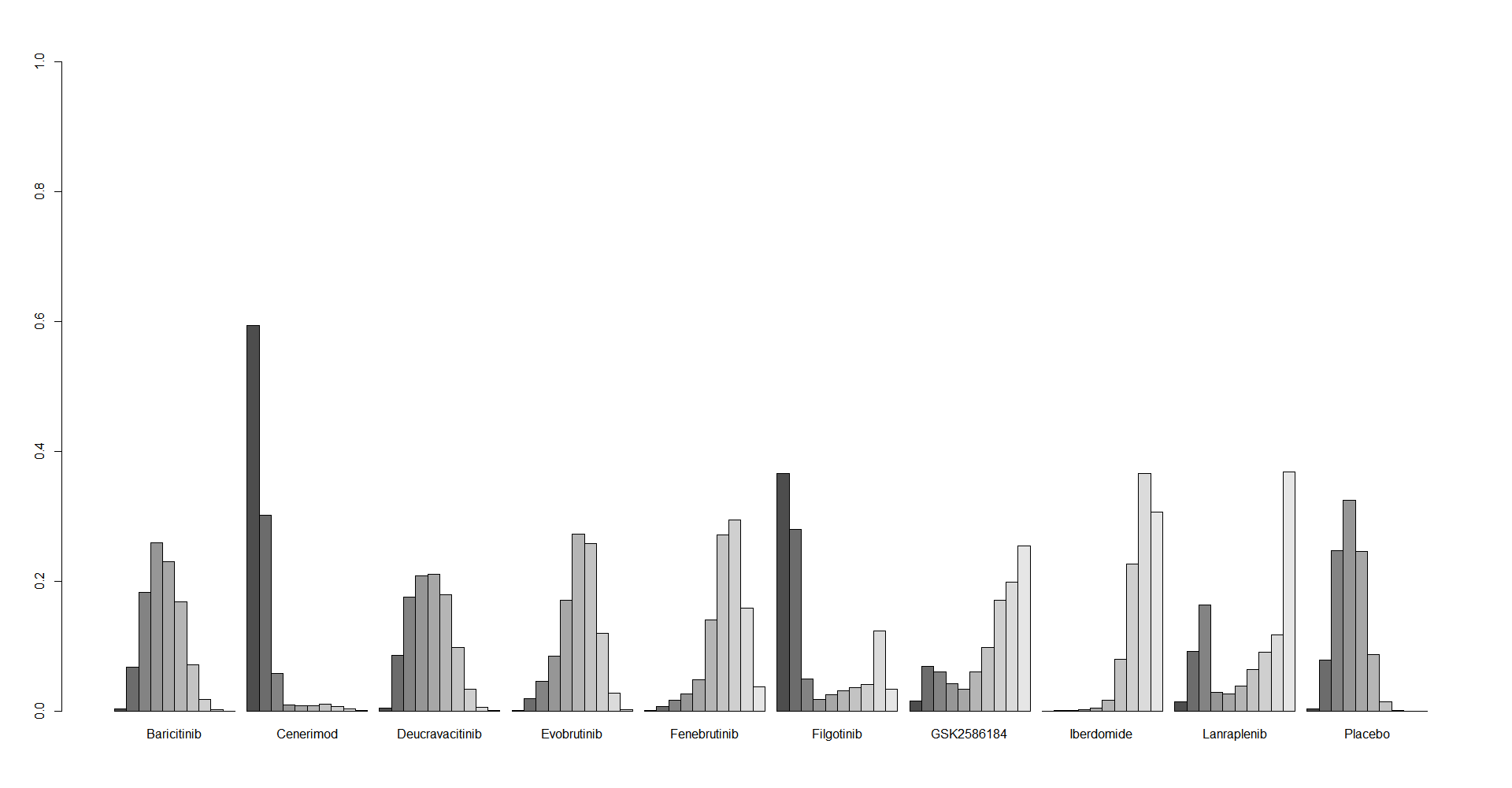
**

adverse reaction

**
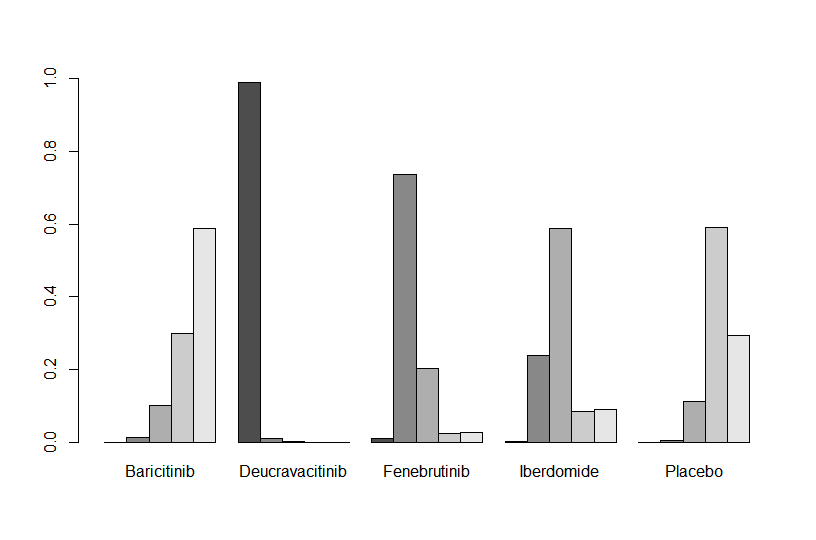
**

CLASI-50

**
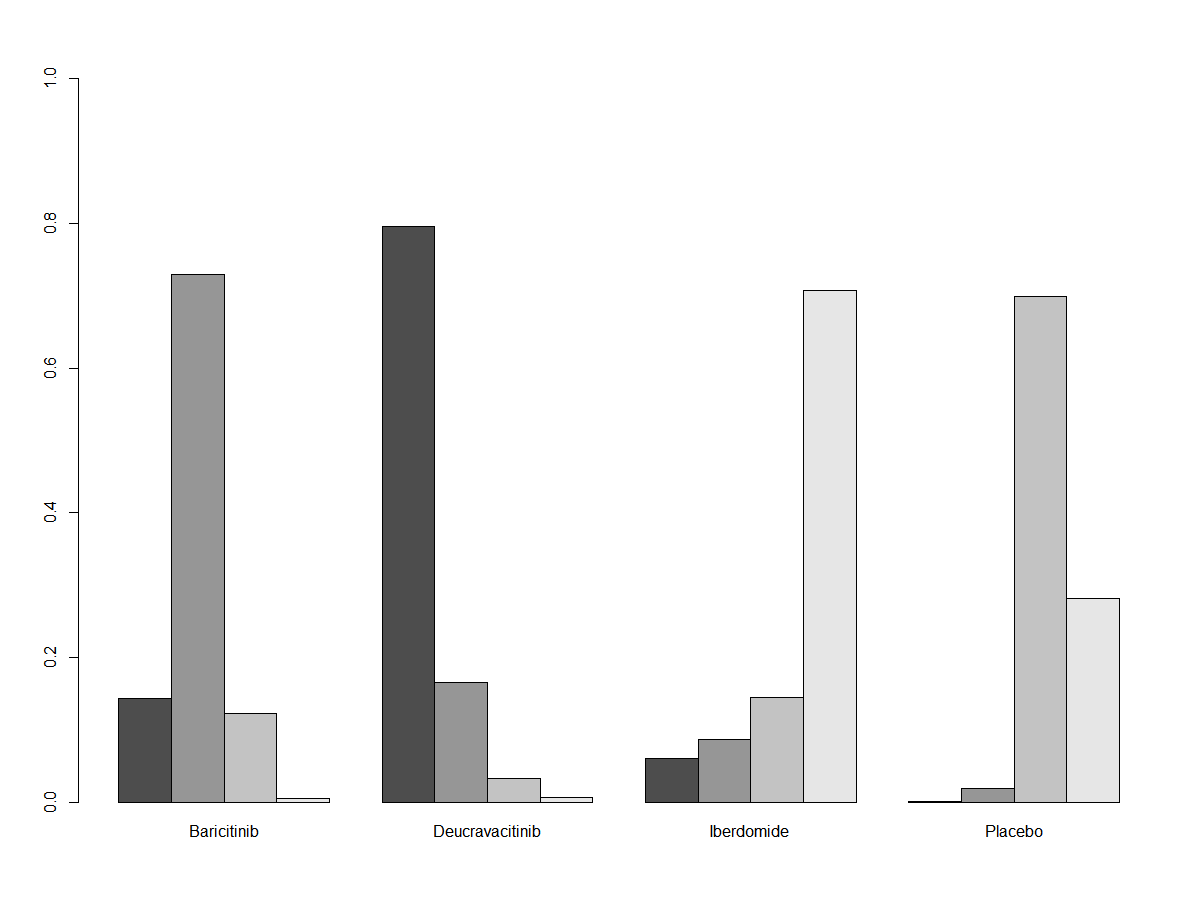
**

tender joint count

**
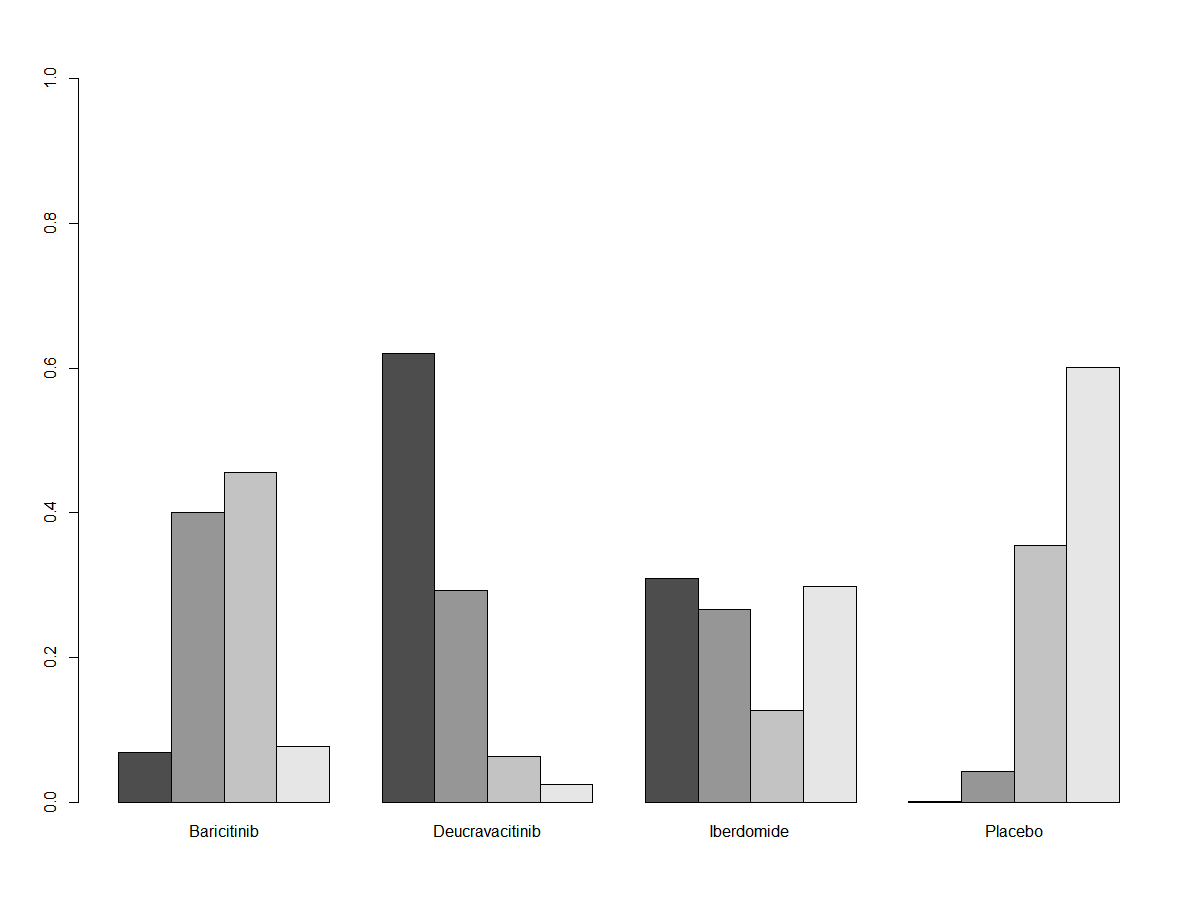
**

swollen joint count
