# Supplementary material for: Efficacy and safety study of targeted small-molecule drugs in the treatment of systemic lupus erythematosus
Source: Arthritis Res Ther. 2024 May 10;26:98. doi: 10.1186/s13075-024-03331-8 (PMC11083747; doi:10.1186/s13075-024-03331-8)
Supplement: Supplementary file 2 — Supplementary Material 2 [file 13075_2024_3331_MOESM2_ESM.doc]

1. CLASI-50

(1) The correlation among each intervention measure

CLASI-50 was reported in 5 studies, including 4 drugs from JAK, BTK and Cereblon. Only drug versus placebo comparisons were reported across these studies, with no pairwise comparisons among all drugs. Direct comparison between Baricitinib and the placebo was made in most studies, and the graph shows that there was no closed loop (see Figure 1).

Figure 1 Network Diagram of Targeted Drug Therapy in the Treatment of SLE Using CLASI-50 as the Outcome Indicator

**(2) Synthesized results**

Deucravacitinib had a significant effect compared with placebo (P < 0.05) (see Figure 2). Some drugs had different effects. The effect of Deucravacitinib was superior to Fenebrutinib (RR = 2.42, 95% CI (1.14, 5.48), P < 0.05) and Iberdomide (RR = 3.04, 95% CI (1.56, 6.55), P < 0.05) (see Table 1). The top three drugs in SUCRA were Deucravacitinib (0.99), Fenebrutinib (0.67), and Iberdomide (0.49) (see Table 4).


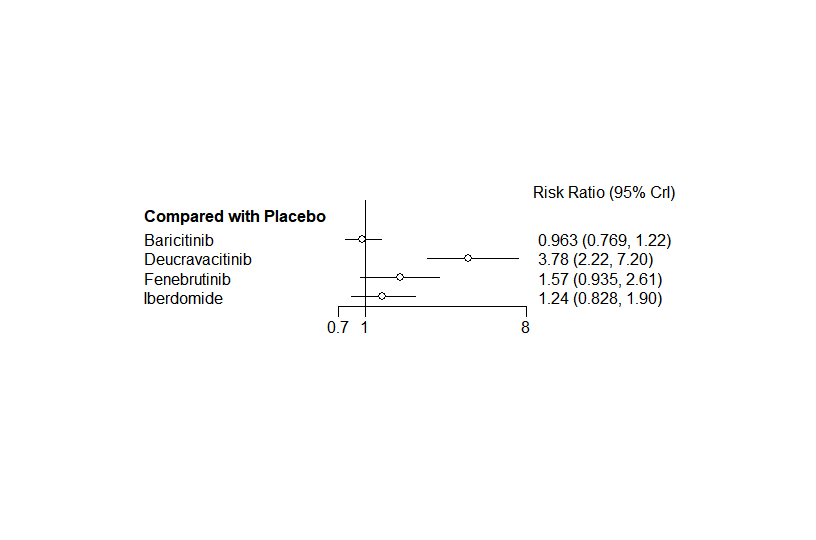


Figure 2 Forest Plot of Meta-analysis of CLASI-50 of Targeted Small-Molecule Drug Therapy Compared with Placebo

Table 1 League Table of CLASI-50 of Targeted Small-Molecule Drug Therapy

| **RR(95%CI)** | | | | | |
| --- | --- | --- | --- | --- | --- |
|  | **Baricitinib** | **Deucravacitinib** | **Fenebrutinib** | **Iberdomide** | **Placebo** |
| **Baricitinib** | 0 |  |  |  |  |
| **Deucravacitinib** | 0.26 (0.13, 0.46) | 0 |  |  |  |
| **Fenebrutinib** | 0.61 (0.35, 1.08) | 2.42 (1.14, 5.48) | 0 |  |  |
| **Iberdomide** | 0.77 (0.48, 1.24) | 3.04 (1.56, 6.55) | 1.25 (0.65, 2.41) | 0 |  |
| **Placebo** | 0.96 (0.77, 1.22) | 3.78 (2.22, 7.20) | 1.57 (0.94, 2.61) | 1.245 (0.83, 1.89) | 0 |

2. tender joint count

(1) The correlation among each intervention measure

Tender joint count was reported in 5 studies, including 3 drugs from JAK and Cereblon. Only drug versus placebo comparisons were reported across these studies, with no pairwise comparisons among all drugs. Direct comparison between Baricitinib and the placebo was made in most studies, and the graph shows that there was no closed loop (see Figure 3).

Figure 3 Network Diagram of Targeted Drug Therapy in the Treatment of SLE Using Tender Joint Count as the Outcome Indicator

**(2) Synthesized results**

Baricitinib and Deucravacitinib had significant effects compared with placebo (P < 0.05) (see Figure 4). There is no statistical difference in the effectiveness of all drugs (see Table 2). The top three drugs in SUCRA were Deucravacitinib (0.92), Baricitinib (0.67), and Placebo (0.25) (see Table 4).


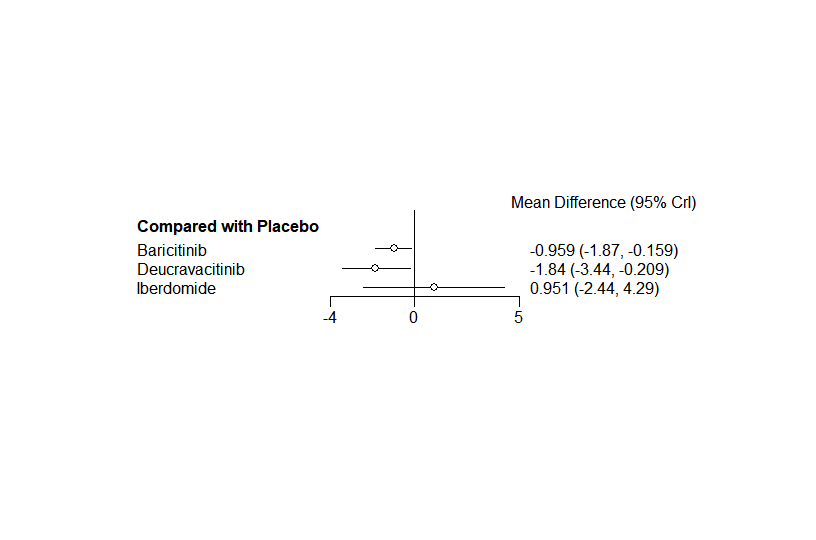


Figure 4 Forest Plot of Meta-analysis of Tender (Swollen) Joint Count of Targeted Small-Molecule Drug Therapy Compared with Placebo

Table 2 League Table of Tender Joint Count of Targeted Small-Molecule Drug Therapy

| MD**(95%CI)** | | | | |
| --- | --- | --- | --- | --- |
|  | **Baricitinib** | **Deucravacitinib** | **Iberdomide** | **Placebo** |
| **Baricitinib** | 0 |  |  |  |
| **Deucravacitinib** | 0.87 (-1.01, 2.64) | 0 |  |  |
| **Iberdomide** | -1.92 (-5.36, 1.56) | -2.8 (-6.44, 0.96) | 0 |  |
| **Placebo** | -0.96 (-1.87, -0.16) | -1.84 (-3.44, -0.21) | 0.95 (-2.44, 4.29) | 0 |

3. Swollen joint count

(1) The correlation among each intervention measure

Tender joint count was reported in 5 studies, including 3 drugs from JAK and Cereblon. Only drug versus placebo comparisons were reported across these studies, with no pairwise comparisons among all drugs. Direct comparison between Baricitinib and the placebo was made in most studies, and the graph shows that there was no closed loop (see Figure 3).

**(2) Synthesized results**

The effects of all drugs were not statistically significant compared to placebo (see Figure 5). There is no statistical difference in the effectiveness of all drugs (see Table 3). The top three drugs in SUCRA were Deucravacitinib (0.84), Iberdomide (0.84), and Baricitinib (0.49) (see Table 4).


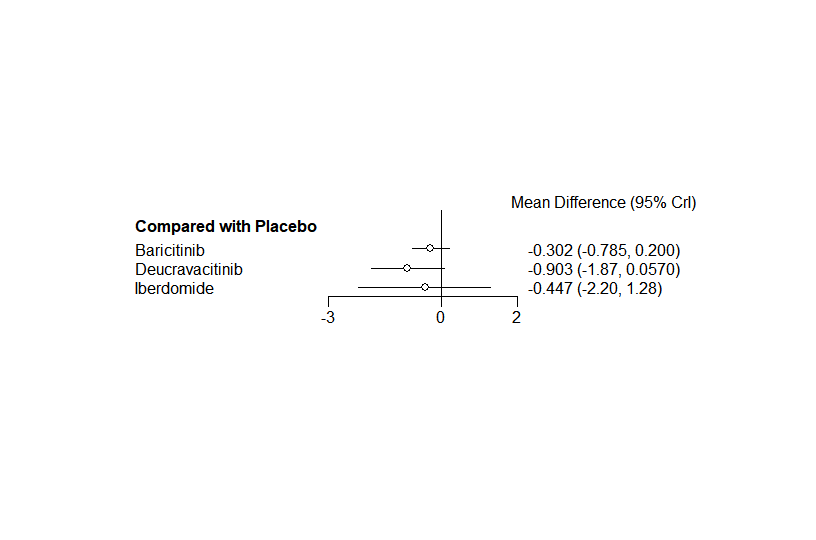


Figure 5 Forest Plot of Meta-analysis of Swollen Joint Count of Targeted Small-Molecule Drug Therapy Compared with Placebo

**Table 3 League Table of Swollen Joint Count of Targeted Small-Molecule Drug Therapy**

| MD**(95%CI)** | | | | |
| --- | --- | --- | --- | --- |
|  | **Baricitinib** | **Deucravacitinib** | **Iberdomide** | **Placebo** |
| **Baricitinib** | 0 |  |  |  |
| **Deucravacitinib** | 0.61 (-0.46, 1.68) | 0 |  |  |
| **Iberdomide** | 0.14 (-1.67, 1.94) | -0.47 (-2.44, 1.54) | 0 |  |
| **Placebo** | -0.29 (-0.78, 0.21) | -0.9 (-1.84, 0.06) | -0.45 (-2.18, 1.31) | 0 |

Table 4 Probability and Ranking of Bayesian Network Meta-analysis of Secondary Outcome Indicators of Each Targeted Small-Molecule Drug

|  | **CLASI-50** |  | **tender joint count** |  | **swollen joint count** |  |
| --- | --- | --- | --- | --- | --- | --- |
|  | **SUCRA** | **No** | **SUCRA** | **No** | **SUCRA** | **No** |
| **Baricitinib** | 0.13 | 5 | 0.67 | 2 | 0.49 | 3 |
| **Deucravacitinib** | 0.99 | 1 | 0.92 | 1 | 0.84 | 1 |
| **Fenebrutinib** | 0.67 | 2 | / | / | / | / |
| **Iberdomide** | 0.49 | 3 | 0.17 | 4 | 0.53 | 2 |
| **Placebo** | 0.21 | 4 | 0.25 | 3 | 0.15 | 4 |

**Meta-regression**

Because the included targeted small-molecule drugs had different doses and courses of treatment, meta-regression was performed on the dose and the course of treatment to discuss the effect of the dose and the course of treatment on results. The results showed that the efficacy of targeted small-molecule drugs was not significantly correlated with the dose and the course of treatment as compared to the placebo, and the results were not statistically significant (see Table 5).

Table 5 Bayesian Network Meta-regression Results of Secondary Outcome Indicators of Each Targeted Small-Molecule Drug

| **Outcome indicators** | **Intervention measures** | **Dose**  **(RR/MD(95%CI))** | **Course**  **(RR/MD(95%CI))** |
| --- | --- | --- | --- |
| **CLASI-50** | Baricitinib | 0.79 (-27.60, 31.11) | 0.57 (-11.06, 9.77) |
|  | Deucravacitinib | -0.07 (-10.74, 11.08) | -0.57 (-15.97, 11.85) |
|  | Fenebrutinib | -0.67 (-3.95, 2.20) | 2.26 (-9.57, 40.26) |
|  | Iberdomide | -1.24 (-62.19, 52.51) | 4.20 (-9.53, 58.54) |
| **tender joint count** | Baricitinib | -1.80 (-6.34, 2.69) | 1.67 (-0.39, 3.77) |
|  | Deucravacitinib | 0.06 (-2.45, 2.58) | 1.56 (-17.41, 42.24) |
|  | Iberdomide | -1.90 (-55.79, 63.88) | -0.22 (-24.20, 23.28) |
| **swollen joint count** | Baricitinib | -0.55 (-3.00, 1.50) | -0.45 (-1.79, 0.78) |
|  | Deucravacitinib | -0.17 (-1.53, 1.11) | 0.45 (-8.28, 15.67) |
|  | Iberdomide | 0.18 (-7.21, 8.69) | 0.64 (-7.61, 14.22) |
